# Recovering European River Invertebrate Communities Homogenize or Differentiate Depending on Anthropogenic Stress

**DOI:** 10.1111/gcb.70716

**Published:** 2026-01-22

**Authors:** Daniela Cortés‐Guzmán, Diana E. Bowler, Marie Anne Eurie Forio, Peter Goethals, Ioannis Karaouzas, Ariane Moulinec, James S. Sinclair, Rudy Vannevel, Peter Haase, Ellen A. R. Welti

**Affiliations:** ^1^ Senckenberg – Leibniz Institution for Biodiversity and Earth System Research Senckenberg Research Institute and Natural History Museum Frankfurt Gelnhausen Germany; ^2^ Biodiversity Monitoring and Analysis UK Centre for Ecology and Hydrology Wallingford UK; ^3^ Department of Animal Sciences and Aquatic Ecology Ghent University Ghent Belgium; ^4^ Institute of Marine Biological Resources and Inland Waters Hellenic Centre for Marine Research Anavyssos Greece; ^5^ Goethe University Frankfurt, Faculty of Biological Sciences Institute of Ecology, Evolution and Diversity Frankfurt am Main Germany; ^6^ Flanders Environment Agency Aalst Belgium; ^7^ Faculty of Biology University of Duisburg‐Essen Essen Germany; ^8^ Great Plains Science Program Smithsonian's National Zoo and Conservation Biology Institute Bozeman Montana USA

**Keywords:** biological traits, community recovery, conservation effectiveness, ecological traits, European rivers, spatial gradients, β‐Diversity

## Abstract

Biodiversity loss can lead to biotic homogenization, whereby local communities within a region become increasingly similar over time, resulting in simplified communities with reduced functionality. However, our understanding of whether alleviating anthropogenic stress can reverse homogenization and promote biotic differentiation (i.e., increasing dissimilarity) remains limited, partly because the effectiveness of conservation actions is often assessed only at the local scale (e.g., increases in local diversity). Here, we examined evidence for biotic differentiation in European river invertebrate communities, a system that has generally shown signs of local recovery. We analyzed 447 time series of river invertebrate communities from 1994 to 2023, spanning 48 river basins across 15 European countries. We then related trends in community similarity within each basin, measured as taxonomic and trait β‐diversity, to spatial gradients of anthropogenic stress, including ecological quality (a proxy of general anthropogenic stress), air temperature increase, and land cover pressure. β‐diversity trends were strongly mediated by anthropogenic stress levels, with communities in lower‐stress basins showing differentiation, while those in higher‐stress basins homogenized. In addition, we found that the direction of β‐diversity change depended less on taxa or traits being gained or lost, and more on the identity of the traits involved, highlighting how trait composition mediates community responses to anthropogenic change. Specifically, additions promoted differentiation at lower stress levels but contributed to homogenization under conditions of higher stress, whereas subtractions exhibited the inverse pattern. Our results demonstrate that β‐diversity responds asymmetrically to spatial variation in anthropogenic stress, with both homogenization and differentiation occurring within a system that is, overall, undergoing recovery. Recognizing the stress‐dependent responses of β‐diversity allows researchers and managers to more accurately assess conservation success and provide recommendations that promote long‐term ecosystem structural and functional recovery.

## Graphical abstract

(a) European river invertebrate communities showed an average increase in local richness over time (1994–2023), indicating partial local recovery following the alleviation of anthropogenic stress (e.g., point‐source pollution). However, this local recovery was not accompanied by increases in β‐diversity, likely due to persistent, unaddressed stressors, such as diffuse pollution or climate change. (b) The levels of remaining anthropogenic stress explained the lack of directionality in average β‐diversity. Communities homogenized (i.e., β‐diversity declined) when stress was still high but differentiated (i.e., β‐diversity increased) when stress levels were lower. (c) The asymmetric response of β‐diversity to anthropogenic stress suggests that, during recovery, communities may initially homogenize and only differentiate once stress levels are sufficiently reduced.

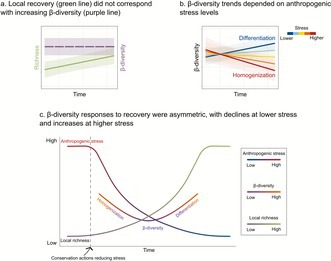

## RESUMEN

La pérdida de biodiversidad puede resultar en homogenización biológica, un proceso en el cual la similitud entre comunidades locales de una región aumenta a lo largo del tiempo, y que a su vez resulta en comunidades simplificadas con funcionalidad reducida. Sin embargo, nuestra comprensión acerca de cómo aliviar estrés antropogénico puede revertir la homogenización y promover la diferenciación biológica (el aumento en disimilitud) permanece limitado, parcialmente porque la efectividad de las acciones para la conservación usualmente se evalúa sólo a escala local (por ejemplo, aumentos en diversidad local). En este estudio, examinamos la evidencia de diferenciación biológica en comunidades de invertebrados en ríos europeos, las cuales han mostrado señales de recuperación a nivel local. Analizamos 447 series de tiempo de comunidades de invertebrados fluviales desde 1994 hasta 2023, que cubren 48 cuencas de drenaje en 15 países europeos. A continuación, relacionamos las tendencias en la similitud entre comunidades en cada cuenca, estimada como diversidad β taxonómica y de rasgos funcionales, con gradientes espaciales de estrés antropogénico, incluyendo calidad ecológica (un proxy de estrés antropogénico en general), incrementos en temperatura del aire y presión por cobertura del suelo. Las tendencias de diversidad β estuvieron fuertemente mediadas por los niveles de estrés antropogénico, con comunidades en cuencas con bajo estrés mostrando diferenciación, mientras que aquellas en cuencas con mayor estrés se homogenizaron. Adicionalmente, encontramos que la dirección del cambio en diversidad β depende menos en que los taxa o rasgos funcionales sean agregados o removidos, y más en la identidad de los rasgos involucrados, resaltando cómo la composición funcional influye en las respuestas de las comunidades al cambio antropogénico. En particular, las adiciones promovieron diferenciación a bajos niveles de estrés, pero contribuyeron a homogenización bajo condiciones de alto estrés, mientras que las sustracciones exhibieron el patrón inverso. Nuestros resultados demuestran que la diversidad β responde de forma asimétrica a la variación espacial en estrés antropogénico, con homogenización y diferenciación ocurriendo en un sistema que esta, en general, recuperándose. Reconocer la dependencia de las respuestas de diversidad β al estrés antropogénico permite a los investigadores y administradores evaluar de forma más precisa los aciertos en conservación, y proveer recomendaciones que promuevan la recuperación a largo plazo de la estructura y función de los ecosistemas.

## 1 Introduction

Biotic homogenization is a major component of biodiversity loss (Blowes et al. 2024; Magurran et al. 2015; Su et al. 2021). Through this process, local communities become more similar over time, reflected in a decrease in β‐diversity (i.e., the similarity in taxonomic or trait composition among local communities within a region). Such increasing similarity often leads to simplified communities with reduced ecological functions and ecosystem services (Biggs et al. 2020; Cavalcante et al. 2023). The opposite process, biotic differentiation, whereby local communities become more different over time and β‐diversity increases, is expected when communities recover and potentially regain lost functions. While homogenization has been observed in freshwater, marine, and terrestrial ecosystems related to declines in regional taxonomic and trait richness (e.g., Blowes et al. 2024; Dornelas et al. 2014, 2019; Karaouzas et al. 2007; Magurran et al. 2015; Petsch 2016; Su et al. 2021), it remains unclear whether differentiation generally occurs in recovering communities. Understanding patterns of differentiation is essential, as many conservation actions aim to recover biodiversity at both local and regional scales.

Recovery describes the process by which biodiversity loss is reversed. In recovering communities, declines in abundance, richness, or diversity are mitigated by reducing or removing their underlying causes, allowing the system to return to a less‐impacted state (Clare et al. 2024; Hutchings et al. 2012; Westwood et al. 2014). Such recovery is often assessed locally, for example, through increases in species richness or functional diversity (e.g., Haase et al. 2023, 2025). However, local increases in diversity alone provide limited insights into the direction of β‐diversity change, as both homogenization and differentiation depend not only on local species changes, but also on how they change in communities across the region. For example, local richness may increase as communities recover, but this could lead to biotic homogenization if the same species increase across all communities (e.g., widespread generalists; Xu et al. 2023). Conversely, communities may become more differentiated without a net gain or even losses in richness if the identities of the species being replaced differ across sites, or if common species are lost in some communities but persist in others (Chase et al. 2019; Hillebrand et al. 2018; Leroy et al. 2024; Tatsumi et al. 2022). Therefore, examining changes in β‐diversity provides essential information on the spatial dynamics of recovery and the mechanisms driving local and regional community change.

Trait‐based approaches offer a powerful and complementary framework to taxonomic metrics for understanding how recovery relates to β‐diversity, as traits often reveal the processes driving β‐diversity patterns. While species replacement may result from either the intensification or alleviation of anthropogenic stress (Cheng et al. 2014; Rolls et al. 2023), trait analyses are key to distinguishing whether such turnover truly reflects recovery. For example, an increase in differentiation driven by the re‐establishment of sensitive or specialist species that were previously filtered out of communities—including those with narrow physiological tolerance, slow life cycles, or weak dispersal abilities (Cavalcante et al. 2023; Gossner et al. 2023; Marr et al. 2013; Rivera et al. 2023; Rumschlag et al. 2023)—may indicate stress release and community recovery following conservation actions. In contrast, persistent or increasing stressors, such as habitat degradation (e.g., Cheng et al. 2014) or climate change (e.g., Magurran et al. 2015; Theodoropoulos and Karaouzas 2021), often lead to homogenization, favoring stress‐tolerant and fast‐dispersing taxa. Trait‐based analyses contribute to our understanding of these dynamics by capturing how changes in community composition affect functions and ecological preferences in response to anthropogenic stress. By examining changes in taxonomic and trait β‐diversity, we can better understand the mechanisms underlying biodiversity change and assess whether reductions in stressors lead to recovery both at local and regional scales.

In river ecosystems, there is growing evidence of general recovery across Europe during recent decades (Bowler et al. 2021; Haase et al. 2023; Outhwaite et al. 2020; Sinclair, Welti et al. 2024). These ecosystems, therefore, provide a unique opportunity to examine whether local recovery generally leads to regional differentiation, an analysis made possible by decades of extensive biomonitoring aimed at assessing ecosystem health (Bonada et al. 2006; Li et al. 2010). The recovery of European rivers is largely attributable to conservation actions that have improved habitat quality by prioritizing pollution reductions required by national and international legislation, such as the Urban Wastewater Treatment Directive or the EU Water Framework Directive (Giakoumis and Voulvoulis 2018; Haase et al. 2025). However, such recovery remains incomplete because the focus on point‐source pollution management has meant that other anthropogenic stressors, such as climate change, pesticides, and diffuse pollution from land use intensification, remain insufficiently addressed (Cortés‐Guzmán et al. 2024; Floury et al. 2013; O'Briain 2019; Voulvoulis et al. 2017; Weisner et al. 2022). As a result, biological communities exhibit a gradient of recovery levels, responding to the alleviation of point‐source nutrient pollution while remaining under pressure from other stressors. This provides an opportunity to ask whether local recovery, albeit partial, leads to regional differentiation or whether ongoing stress limits differentiation despite increases in local diversity.

We analyzed trends in taxonomic and trait β‐diversity among 447 river invertebrate communities (local scale) from 48 basins (regional scale) across 15 European countries from 1994 to 2023 to determine whether communities have differentiated, consistent with patterns expected under observed local recovery (Haase et al. 2023; Sinclair, Welti et al. 2024). We related temporal trends in taxonomic and trait β‐diversity to spatial stress gradients in (i) ecological quality, used as a proxy for pollution and general anthropogenic stress, (ii) intensity of air temperature warming, and (iii) land cover pressure. We hypothesized that taxonomic and trait β‐diversity have, on average, increased over time, indicating differentiation, but that these trends are stronger in basins with lower stress levels along the spatial gradients. Our findings contribute to identifying the mechanisms and drivers of biodiversity change at regional scales, informing biodiversity recovery and guiding targeted conservation actions aiming at supporting ecosystem functions and services.

## 2 Methods

### 2.1 Biological Data

We obtained river invertebrate data from Welti et al. (2024) and Sinclair, Stubbington, et al. (2024). To expand geographic and temporal coverage, we complemented the dataset with additional data obtained through direct requests to ecologists and environmental managers from Belgium, Germany, and Greece. These newly available data filled gaps for previously missing countries and met the data requirements regarding sampling methods and taxonomic resolution detailed below.

To assess temporal changes in β‐diversity among river invertebrate communities, we used river basins as the spatial unit of analysis, as communities within the same basin are hydrologically connected and thus form a metacommunity (Tonkin et al. 2018). We selected groups of sampling sites that belong to the same basin, based on the European catchments and river network system Ecrins (https://www.eea.europa.eu/en/datahub/datahubitem‐view/a9844d0c‐6dfb‐4c0c‐a693‐7d991cc82e6e). To ensure consistent sampling methods and taxonomic resolution among sites within each basin, we restricted site selection to those located in the same country and from the same data provider, given that some river basins can span multiple countries. Additionally, to ensure consistent temporal comparisons among sites, we selected the same sampling years for all sites within each basin. Most data were collected by kick‐sampling and represent, as far as possible, community‐level sampling of all invertebrates. Abundance data were recorded for each sampled taxon. Our final dataset consists of 447 time series of river invertebrates sampled once a year over at least 5 years during the same season (i.e., any three consecutive months) across 48 basins spanning 15 European countries. Basins contain between 5 and 32 sites (median = 8 sites) and have 5 to 20 sampling years (median = 10 years) between 1994 and 2023.

In addition, the ecological quality ratio (EQR) was available for all selected sites. The EQR is a standardized index required by the EU Water Framework Directive (WFD 2000) to assess the ecological status of water bodies. The EQR compares observed communities to least‐impacted reference communities in each region, based on the composition of sensitive and tolerant taxa and other multi‐metric indices. Values typically range from close to 0 to more than 1, with 0 representing low similarity to reference communities and thus higher impacts and 1 or higher representing high similarity and thus lower impacts. Although the specific metrics used in the EQR calculations can vary across countries, the result is a standardized measure that ensures comparability among freshwater ecosystems exposed to different anthropogenic stress.

### 2.2 Trait Composition

To assess changes in trait composition of invertebrate communities, we extracted information on biological and ecological traits from freshwaterecology.info (Schmidt‐Kloiber and Hering 2015). Biological traits are inherent morphological and life‐history features measured at the individual or taxonomic‐group level, without reference to the environment (Violle et al. 2007), and thus reflect homogenization or differentiation in ecosystem processes and functions (Dawson et al. 2021; Violle et al. 2007). We analyzed six biological traits: aquatic stages, dispersal strategy, number of reproductive cycles per year (i.e., voltinism), feeding habits, resistance forms, and maximum body size (Table S1). Ecological traits are descriptors of habitat use (Usseglio‐Polatera et al. 2000); therefore, homogenization of ecological traits reflects increased similarity in physical or chemical habitat conditions or biotic preferences. We examined three ecological traits: temperature preference, microhabitat preference, and saprobity (i.e., sensitivity to organic matter contamination).

Trait information was extracted for each taxon and covered 35%–77% of taxa at the provided level. When trait information at the provided level was unavailable, we imputed missing values using information from the next higher taxonomic rank, for example, genus‐level traits when species‐level data were missing, or family‐level traits when genus‐level data were unavailable. If this information was also missing, we used the average trait value across all taxa belonging to the same higher‐level group, for example, average across all species within the same genus. Taxa without available trait information after this process were removed from subsequent trait analyses. This process resulted in a final coverage of 72%–93% for biological traits and 71%–90% for ecological traits, varying in coverage with individual traits. This approach is based on the assumption that using mixed‐level trait information is a reliable approach since closely related taxa have similar traits (Burns and Strauss 2011), and it has better performance than omission or other imputation methods (Johnson et al. 2021; Palacio et al. 2022; Poyatos et al. 2018).

We ran all analyses in R 4.3.2–4 (R Core Team 2024). To calculate community trait composition, we first transformed the values from freshwaterecology.info into proportions to allow for comparisons between different coding systems (e.g., 10‐point assignment, fuzzy coding, or single assignment). Specifically, for traits represented by multiple columns (indicating affinity to multiple modalities), the values in each row were divided by the row sum across those columns, so that values within each trait represent comparable proportions that all sum to 1. Then, the trait proportions were used to compute the mean trait value for each trait and community as a ‘community weighted mean’ (CWM; following Lavorel et al. 2008), calculated using relative abundances and the function *functcomp* from the “FD” package (Laliberté et al. 2014). To obtain an even distribution of traits with different numbers of modalities, we weighted the contribution of each trait to the CWMs using weights calculated with the function *gawdis* from the “gawdis” package (De Bello et al. 2021).

### 2.3 Evidence of Local Recovery

To support our assumptions of local recovery, we evaluated trends in local taxonomic and trait richness over time within the investigated basins. Taxonomic richness represents the total number of taxa at each site and year. Trait richness represents the volume of multidimensional trait space occupied by all taxa in a community. Biological and ecological trait richness were calculated using a dissimilarity matrix based on the weighted traits and taxa relative abundances, employing the *dbFD* function from the “FD” package (Laliberté et al. 2014). To assess temporal trends, we modeled taxonomic and trait local richness as response variables, using year as a predictor and including a random intercept for site. To evaluate whether local trends generally increase or decrease within basins, we aggregated local trends by including a random slope for basin in the models. The overall slope estimated from these models represents the average across all sites, and the estimated slopes for each basin represent the average trend across sites within a basin. Finally, we examined the distribution of local trends aggregated per basin in relation to ecological quality, temperature trends, and land cover pressure (see below) to identify potential patterns of local diversity along anthropogenic stress gradients.

### 2.4 β‐Diversity Indices

We calculated β‐diversity among sites in the same basin, based on the composition of taxonomic, biological, and ecological traits (Table S1). For each sampling year and basin, we calculated multiple‐site β‐diversity indices using the Bray–Curtis dissimilarity index. We used taxonomic composition matrices and CWM matrices for biological and ecological traits to calculate the indices, using the function *beta.multi.abund* from the “betapart” package (Baselga 2017). This function standardizes variation in abundance to be independent of the number of sites and not mathematically constrained by the number of species (Baselga 2017), ensuring that β‐diversity values are comparable between basins with different numbers of sites and taxon richness.

### 2.5 Temperature and Land Cover Data

Warming was represented as the change in annual basin temperatures through time. We obtained daily mean air temperature values for each site from the E‐OBS dataset and the Copernicus Climate Change Service (EU‐FP6 project UERRA, https://www.uerra.eu; https://surfobs.climate.copernicus.eu/surfobs.php; Cornes et al. 2018) with a resolution of 0.1°. Daily temperature data were extracted from 1993 to 2023 using the geographical coordinates of the sampling sites and calculated the mean for the sampling months, averaging across all sites within each basin. To estimate temperature trends, we performed a linear regression of temperature over time for each basin and used the slope as an indicator of warming over the sampling period, with higher values representing higher levels of stress.

Land use pressure represented the composition of forest (assumed lower pressure) and urban/agricultural land cover (assumed higher pressure) for each basin averaged across sites and years. We obtained land cover data from the Copernicus Climate Change Service, Climate Data Store (2019) database, with a horizontal resolution of 300 m. From this dataset, we extracted values for three dominant types: forested areas (including tree cover and mosaic trees and shrubs), urban areas, and agricultural areas (including croplands and mosaic croplands). These categories represented over 80% of land cover for most of the sites; therefore, other types such as grasslands, mosaic vegetation, and sparse vegetation were excluded from the analyses. For each sampling year, we calculated the proportion of each land cover type within the upstream area of each site. The average proportions across sampling years and sites within each basin were used to obtain a spatial mean land cover composition per basin.

Forest cover was strongly negatively correlated with agricultural cover (*r* = −0.89) and moderately negatively correlated with urban cover (*r* = −0.40). To account for these correlations among land cover types, we conducted a principal components analysis (PCA) and used the first two principal components (explaining 96.5% of the variance) as indicators of land cover pressure in our analyses. The first component (PC1) represents a gradient of increasing forest cover and decreasing agricultural and urban cover, while the second component (PC2) captures increasing urban cover.

### 2.6 β‐Diversity Trends

As β‐diversity is a dissimilarity index, decreases in β‐diversity represent homogenization, while increases in β‐diversity suggest differentiation. To estimate the across‐basin temporal trend in β‐diversity, we modeled taxonomic, biological, and ecological trait β‐diversity over time with a random intercept for basins using functions from the package “glmmTMB” (Brooks et al. 2024). The form of the model was: glmmTMB (β‐diversity~year + (1|basin), family = beta_family(link = “logit”)). To estimate the trend for each basin, we ran a second model with the form: glmmTMB (β‐diversity~basin + basin:year, family = beta_family(link = “logit”)).

We also estimated the effects of three anthropogenic drivers on β‐diversity trends. Ecological quality was used as a proxy for general anthropogenic stress, temperature trends indicated the intensity of warming, and the PCA land cover components indicated land cover pressure. To incorporate EQR as a spatial variable, we averaged values across sites and years within each basin, with higher values indicating lower levels of anthropogenic stress. These three drivers were examined as spatial variables across basins representing gradients of stress (Figure S1). We used mixed models to relate taxonomic, biological, and ecological trait β‐diversity over time to the mean EQR, temperature slope, and land cover PC axes, including a random intercept for basins. Before running the models, stressor variables were scaled (value−mean/SD) to ensure that the coefficients were comparable. Each driver was examined in separate models that included an interaction with sampling year to test whether spatial gradients in anthropogenic stress could explain differences in β‐diversity trends. The form of each model was: glmmTMB (β‐diversity~driver × year + (1|basin), family = beta_family(link = “logit”)).

To assess the potential influence of local diversity trends on β‐diversity patterns, we re‐analyzed the results by repeating the mixed models after excluding basins that, on average, exhibited decreasing trends in local richness. Our goal was to isolate patterns associated with recovery, so only those basins where local communities showed increasing richness over time were retained. Specifically, for each β‐diversity model—taxonomic, biological, and ecological traits—basins were excluded based on the corresponding type of local richness. We did not analyze degrading basins separately due to their limited number (15 basins or fewer), which constrained statistical power and would have reduced the robustness of any conclusions.

The model residuals were assessed for spatial and temporal autocorrelation and for heterogeneity of variance using both visual diagnostics and formal tests. The analyses did not detect residual autocorrelation or variance heterogeneity (Figure S2).

### 2.7 Additive and Subtractive Components of β‐Diversity Change

To determine whether changes in β‐diversity were driven by taxon or trait increases in abundance, hereafter ‘additions’, or taxon or trait declines in abundance, referred to as ‘subtractions’, we partitioned the temporal change in taxonomic, biological, and ecological trait β‐diversity for each basin into its additive and subtractive dynamic components following methods from (Tatsumi et al. 2021, 2022). This approach quantifies the relative contribution of taxon/trait additions and subtractions to homogenization and differentiation (negative or positive change in β‐diversity, respectively). Furthermore, we also quantified the additive and subtractive components of individual traits to trait β‐diversity (following Tatsumi et al. 2021, 2022) to identify which individual traits were driving these changes.

We then evaluated how additions and subtractions contributed to changes in β‐diversity along anthropogenic stress gradients. To do so, we analyzed relationships between the additive and subtractive components with ecological quality, temperature trends, and land cover pressure. Basins were included as a random intercept, assuming a Gaussian distribution. Additionally, to identify which specific traits significantly contributed to changes in β‐diversity, we tested whether trait‐level changes due to additions and subtractions differed significantly from zero across years, using individual trait changes as response variables, basins as a random intercept, and a Gaussian distribution.

## 3 Results

### 3.1 Trends in Taxonomic, Biological, and Ecological Trait Richness

**FIGURE 1:**
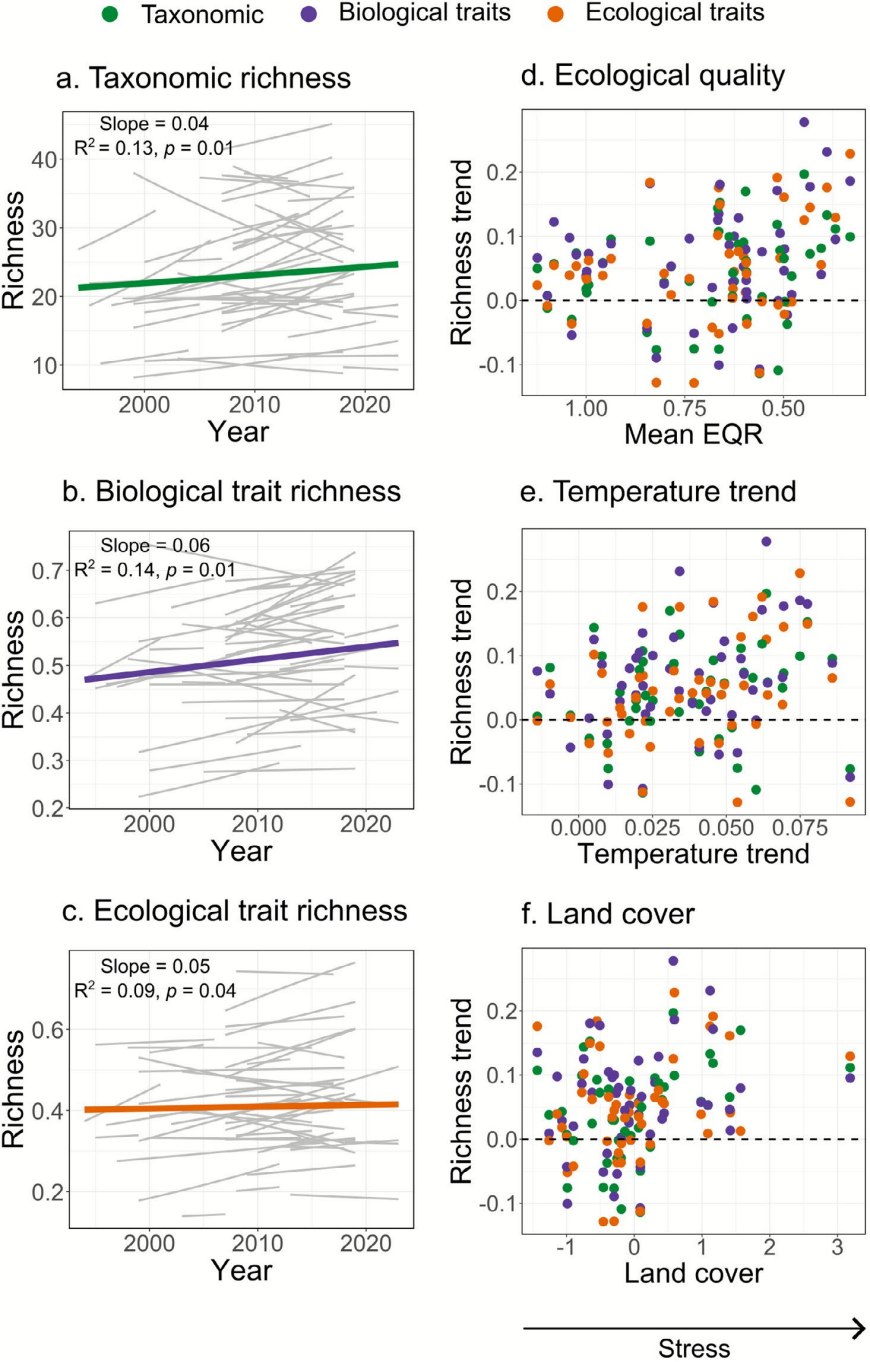
Temporal trends in local (a) taxonomic, (b) biological, and (c) ecological trait richness, grouped by basin. In (a–c), grey lines represent trends for individual basins and colored lines represent the average predicted trend across all sites. The estimated year slope, *R* ^2^, and corresponding *p*‐values are shown in each panel. In (d–f), the estimated slope values per basin across spatial gradients of (d) ecological quality, (e) temperature trend, and (f) land cover (PC2 gradient representing increasing urban cover) are shown.

To examine whether local recovery is reflected in regional differentiation or whether ongoing stress limits differentiation despite increases in local diversity, we first analyzed trends in local richness within basins. Across all sites, local taxonomic, biological, and ecological trait richness increased over time (colored lines in Figure 1), supporting a general trend of recovery. However, trends varied markedly among basins (grey lines in Figure 1), with some showing increases and others declines. Notably, these trends showed no clear relationship with anthropogenic stress gradients, indicating that both recovering and degrading communities occurred throughout the entire stress gradient.

### 3.2 Trends in Taxonomic, Biological, and Ecological Trait β‐Diversity

**FIGURE 2:**
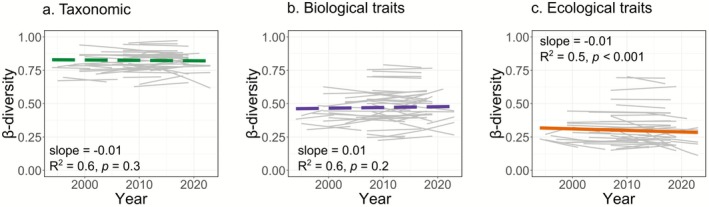
Temporal trends in β‐diversity based on (a) taxonomic composition, (b) biological traits, and (c) ecological traits in 48 European basins. Grey lines represent trends for individual basins and colored lines the average predicted trend across basins. Increasing trends indicate differentiation, whereas decreasing trends indicate homogenization. Estimated slopes, *R* ^2^, and *p*‐values are shown for each model.

To assess whether communities have differentiated in a system showing overall recovery—albeit with substantial individual variation—we analyzed temporal trends in β‐diversity across basins. Our analyses showed highly variable β‐diversity trends among basins across all metrics, with individual basins showing both increases and decreases (Figure 2 and Figure S3). As a result, average trends lacked a strong direction. We found slight but significant mean homogenization in ecological trait β‐diversity (Figure 2c), meaning that the communities became, on average, more similar over time (10.9% decrease in 30 years, 0.36% per year). However, taxonomic and biological trait β‐diversity did not show significant temporal trends. Moreover, only a few basin slopes differed significantly from zero (Figure S4).

### 3.3 Effect of Anthropogenic Stress on β‐Diversity Trends

**FIGURE 3:**
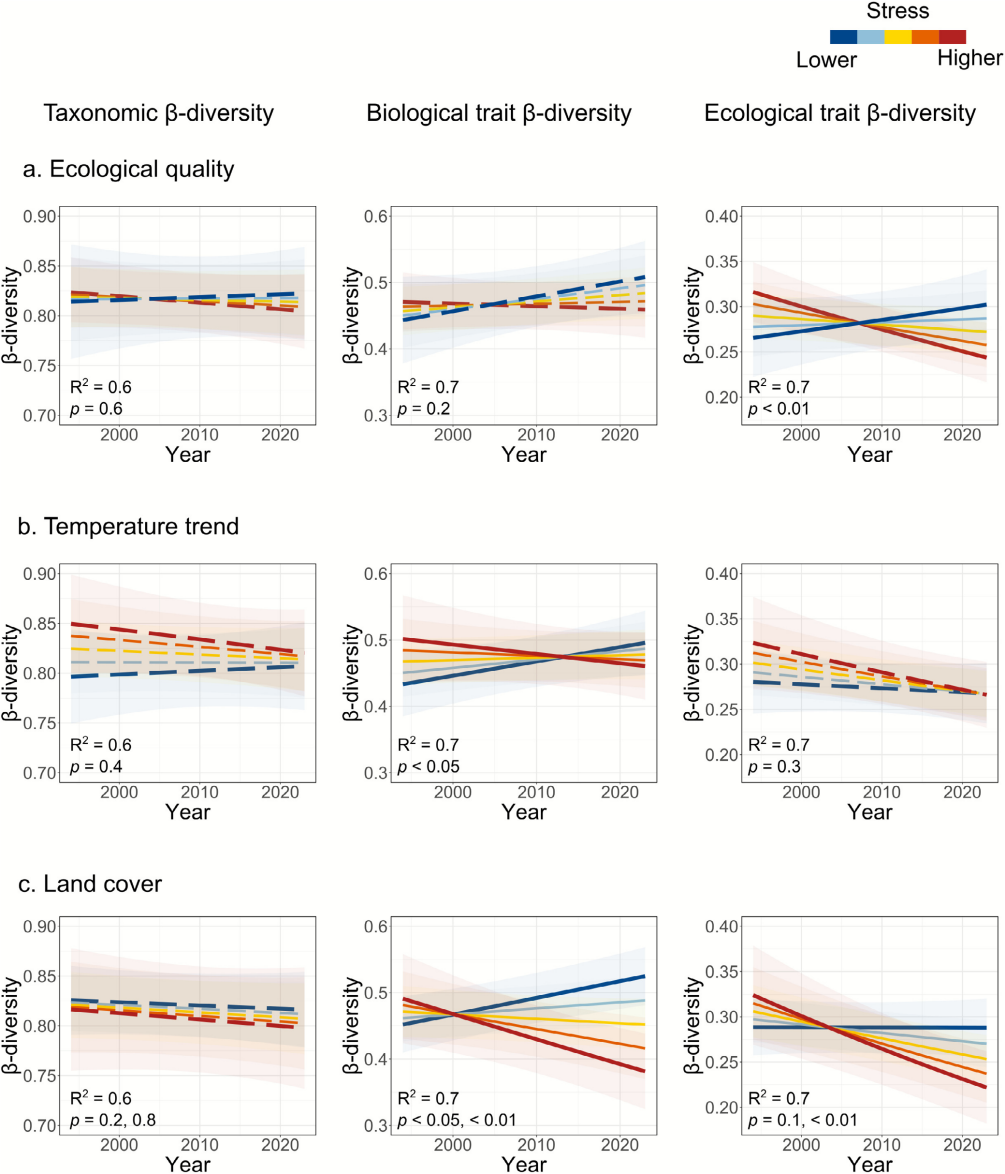
Predicted effects of (a) ecological quality, (b) temperature trends, and (c) land cover on long‐term trends in taxonomic (left panels), biological (middle panels), and ecological (right panels) trait β‐diversity from generalized linear mixed models. *R* ^2^ and *p*‐values are shown for each model; for land cover pressure, *p*‐values correspond to PC1 and PC2. Line colors represent predicted trends across gradients of anthropogenic stress, ranging from lower stress (higher average EQR, lower temperature slope, or lower urban/higher forest cover) to higher stress (lower average EQR, higher temperature slope, or higher urban cover). Stress levels were represented by five equally spaced values along the range of each variable. For land cover, we selected values along the PC2 range while keeping PC1 constant. Solid lines represent significant interactions and dashed lines represent non‐significant interactions. Decreasing trends indicate homogenization, and increasing trends indicate differentiation.

To explore the mechanisms behind this heterogeneity in responses, we analyzed how trends in β‐diversity related to spatial gradients of anthropogenic stress (Figure 3). Communities tended to differentiate only in basins with lower stress levels. Specifically, basins with higher ecological quality, lower temperature slopes (i.e., low or moderate warming), and lower land cover pressure (i.e., lower urban and agricultural cover, and higher forest cover) tended to differentiate, with similar relative importance among drivers (1%–3% β‐diversity change per 1 SD in the driver; see full model equations in Table S2). In contrast, a gradual shift toward community homogenization was associated with lower ecological quality, higher temperature trends (i.e., more intense warming), and more intense land cover pressure (i.e., higher urban and lower forest cover). Furthermore, β‐diversity was generally initially higher and decreased over time in basins showing higher stress, in comparison to basins with less stress, where β‐diversity was initially lower and increased over time (Figure 3).

When analyzing only recovering communities (based on trends in local richness), we found similar β‐diversity patterns (Figure S5 and Table S3). These results suggest that *recovering* communities tended to homogenize in basins with higher stress, whereas *recovering* communities differentiated only in basins with lower stress. Furthermore, trends in local richness were not correlated with β‐diversity trends (Figure S6), indicating that local declines or increases in richness do not necessarily translate into regional‐scale patterns of homogenization and differentiation, respectively.

**FIGURE 4:**
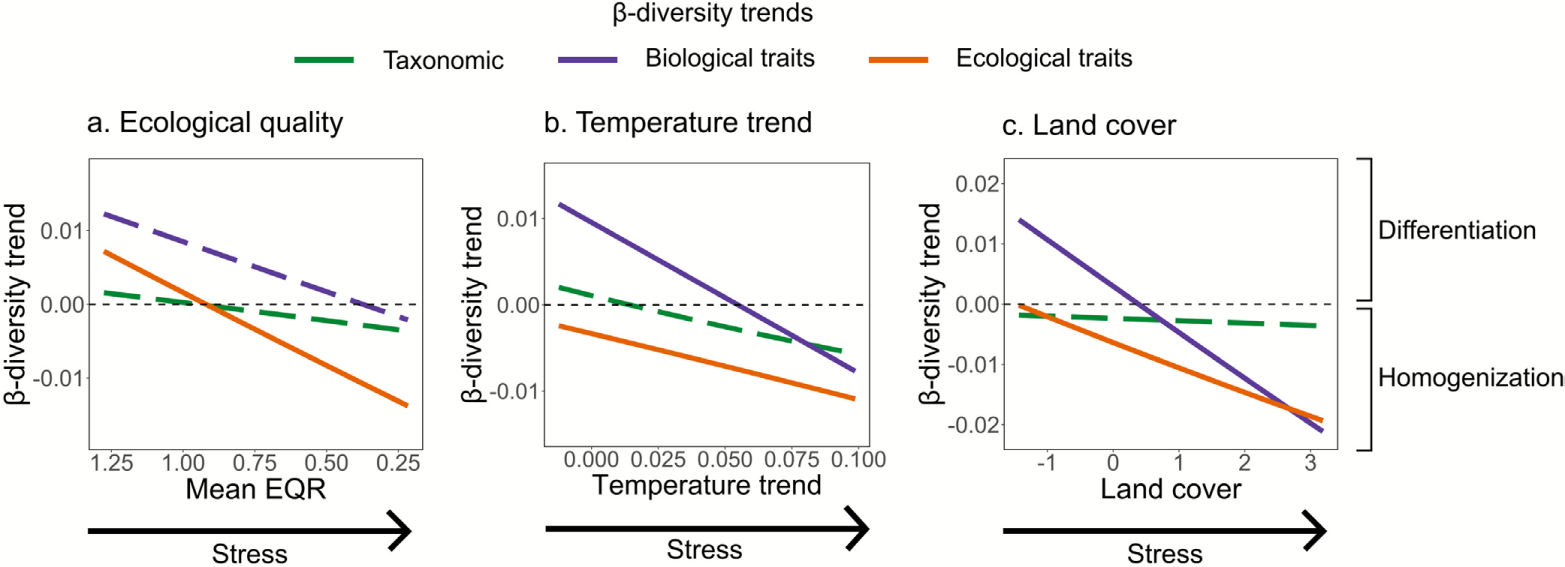
Marginal effects of year on β‐diversity along spatial gradients of (a) ecological quality, (b) temperature trend, and (c) land cover (PC2 gradient while keeping PC1 constant). Solid lines represent significant interactions with the sampling year, and dashed lines represent non‐significant interactions. Negative β‐diversity trends indicate homogenization, and positive trends indicate differentiation.

We further examined how the direction and magnitude of β‐diversity trends varied along the spatial gradient of anthropogenic stress to highlight potential tipping points in environmental conditions where the trend in β‐diversity shifts from homogenization to differentiation, or vice versa. The magnitudes of taxonomic β‐diversity trends were generally non‐significant and narrower in range compared to those of biological and ecological trait β‐diversity (Figure 4). This suggests that shifts in taxonomic composition along spatial gradients of anthropogenic stress may be subtle or relatively harder to detect. In contrast, trends in biological and ecological trait β‐diversity exhibited greater variability. Notably, ecological trait β‐diversity trends were mostly negative, with a shift toward positive trends occurring only at the lower stress levels, particularly for temperature trends and land cover pressure.

### 3.4 Mechanisms Driving β‐Diversity Change: Taxa and Trait Additions and Subtractions

**FIGURE 5:**
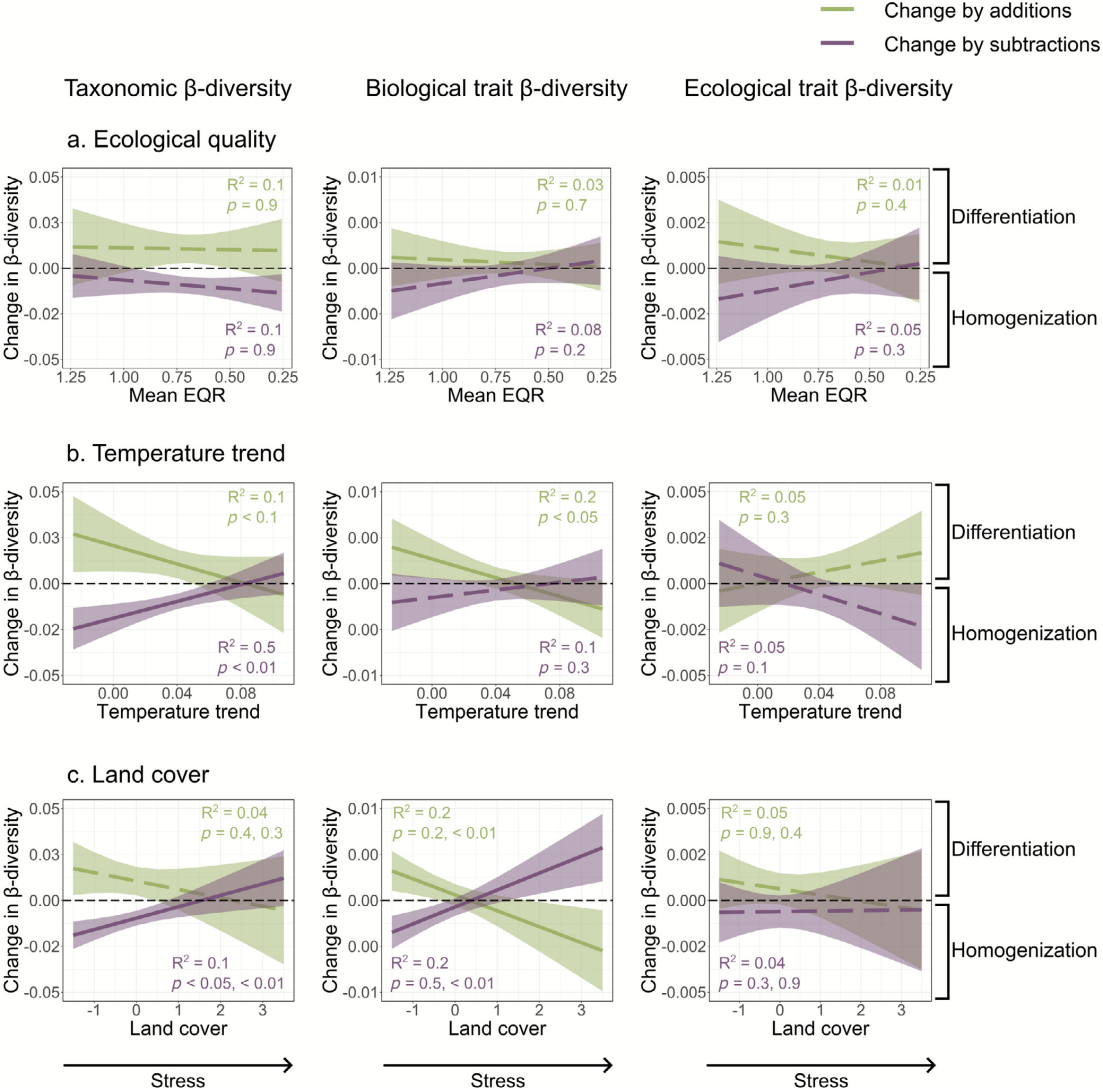
Contribution of subtractive and additive dynamic components to the temporal change in β‐diversity along spatial gradients of (a) ecological quality, (b) temperature trend, and (c) land cover pressure. *R* ^2^ and *p*‐values from generalized linear mixed models are shown for each model; for land cover pressure, *p*‐values correspond to PC1 and PC2. Solid lines represent significant interactions with the sampling year, and dashed lines represent non‐significant interactions.

We examined how the addition and subtraction of taxa and traits influenced temporal changes in β‐diversity along spatial gradients of anthropogenic stress. In general, the addition of taxa and biological traits led to differentiation at lower temperature slopes and land cover pressure, but resulted in homogenization under higher intensities of these same stressors (Figure 5). However, the explained variance of the models was relatively low (20% or less), suggesting high variability in the responses of additions and subtractions to stress gradients. Conversely, subtraction of taxa and biological traits tended to promote homogenization at lower stress levels, but led to differentiation as stress intensified. Along the ecological quality gradient, additions and subtractions of taxa, biological, and ecological traits did not show significant patterns. Similarly, changes of ecological traits did not produce clear directional changes in β‐diversity; instead, both mechanisms—additions and subtractions—could lead to differentiation or homogenization.

**FIGURE 6:**
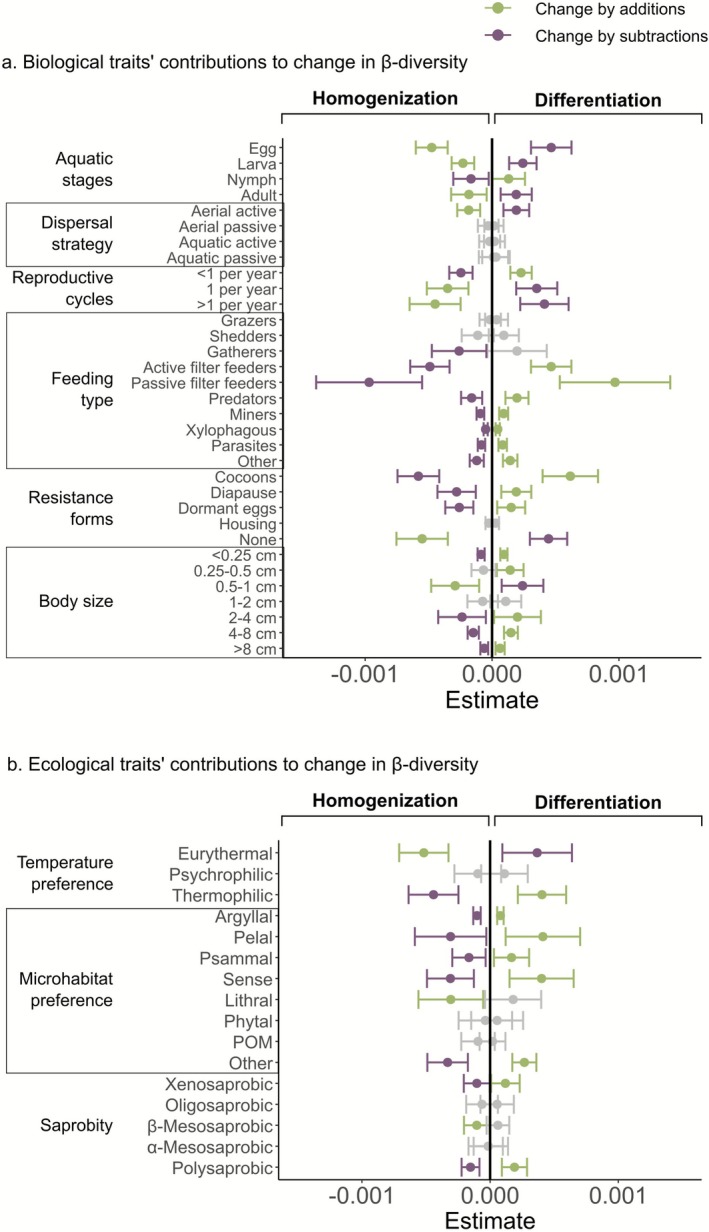
Contributions of (a) biological and (b) ecological traits to the temporal change in β‐diversity by additions and subtractions across basins. Dots represent the intercept of models testing a difference from 0; error bars indicate 95% confidence intervals. Green symbols represent significant change by additions, purple symbols represent significant change by subtractions, and grey symbols represent non‐significant change.

To determine which specific traits drove differentiation and homogenization, we examined the relative contribution of individual traits to temporal changes in β‐diversity. Differentiation primarily resulted from adding taxa with faster life cycles and aquatic nymphs (e.g., Ephemeroptera, Trichoptera, Plecoptera), specialists (i.e., taxa with specialized resistance forms), and by increases in feeding groups except for grazers, shredders, and gatherers, and body size categories except for 0.5–1 cm (Figure 6). Differentiation was also caused by subtractions of tolerant taxa, for example, those with aquatic eggs, larvae, and adults, but not nymphs or pupae, like Coleoptera (Dytiscidae, Hydrophilidae, Elmidae) or non‐insect taxa with slower life cycles, such as Hirudinea, Oligochaeta, and Crustacea. Regarding ecological traits, differentiation resulted from additions of warm‐tolerant taxa (thermophilic), with a preference for small to medium grain‐size microhabitats, and taxa that tend to occur in unpolluted (xenosaprobic) and heavily polluted waters (polysaprobic; Figure 6).

The opposite set of traits characterized communities undergoing homogenization, including subtractions of taxa with faster life cycles, specialized resistance forms, and warmer thermal preferences, and additions of taxa with slower life cycles, without specialized resistance forms, small body sizes (0.5–1 cm), broad thermal tolerance (eurythermal), and occurring in moderately polluted waters (β‐mesosaprobic).

## 4 Discussion

Although European river invertebrate communities have generally shown signs of recovery over the last several decades (Figure 1; Haase et al. 2023; Lyche Solheim et al. 2025; Sinclair, Stubbington, et al. 2024), β‐diversity trends varied along spatial gradients of anthropogenic stress. Communities differentiated in basins with higher ecological quality, low to moderate warming, or lower urban cover, whereas communities homogenized in basins with lower ecological quality, intense warming, or higher urban cover. Moreover, the similar response observed in recovering communities (Figure S5) suggests that this pattern reflects the alleviation of some stressors but the persistence of others. As a result, European river communities show only partial recovery, a trend that mirrors patterns reported for other taxonomic groups and ecosystems (e.g., Bowler et al. 2021; Cano‐Barbacil et al. 2025; De Los Santos et al. 2019; Outhwaite et al. 2020). This asymmetric community response explains the lack of significant overall trends for taxonomic and biological trait β‐diversity, and only a weak trend of homogenization for ecological trait composition. This dependency of trends on spatial stress levels might further elucidate why global syntheses of β‐diversity change in freshwater, marine, and terrestrial communities often report mixed outcomes (Blowes et al. 2024; Dornelas et al. 2019; Rolls et al. 2023). Our study expands on such previous investigations by linking differentiation and homogenization responses to intensities of anthropogenic stress, despite a general background of recovery.

Basins exposed to higher levels of anthropogenic stress tended to show higher initial β‐diversity than basins exposed to lower spatial stress levels. This contrasts with many studies in terrestrial and aquatic ecosystems, which typically report lower β‐diversity under high anthropogenic stress due to habitat homogenization (e.g., Agra et al. 2023; Chase 2007; Davey et al. 2012; Dormann et al. 2007; Ekroos et al. 2010; Magurran et al. 2015). However, several mechanisms may explain the initially higher β‐diversity in basins with higher stress. First, these basins showed greater initial heterogeneity in ecological quality among sites (EQR s.d. = 0.22 vs. 0.13 in lower‐stress basins), suggesting a mix of degraded and relatively undisturbed conditions. Such patchiness is common in highly impacted systems where stressors might be unevenly distributed or act unevenly in communities across space, resulting in greater environmental heterogeneity, which can increase β‐diversity (Astorga et al. 2014; Daleo et al. 2023; Hawkins et al. 2015). Second, high β‐diversity under high anthropogenic stress can also result from strong environmental filtering (Chase 2003; Leibold et al. 2004). Although environmental filtering usually leads to homogenization *within* sites over time, it can initially increase β‐diversity if different stressor types or intensities dominate at different sites, selecting for different subsets of tolerant taxa (Huber et al. 2020; Liang et al. 2025). In other words, environmental filtering can increase heterogeneity *across* communities, and therefore β‐diversity, when the environmental pressures vary spatially. Despite initially higher β‐diversity, as recovery progresses, stressor levels become more similar across more‐ and less‐impacted sites, potentially leading to homogenization, regardless of whether the previously high β‐diversity was a result of high heterogeneity, strong filtering, or both.

Our findings reveal important differences in how taxonomic, biological, and ecological trait β‐diversity respond to spatial gradients of anthropogenic stress, offering insight into potential tipping points in biodiversity change. Taxonomic β‐diversity trends were generally weak and of lower magnitude, suggesting subtle or less detectable shifts in taxon composition. In contrast, patterns in biological and especially ecological trait β‐diversity were more pronounced, suggesting changes in trait composition may precede detectable shifts in taxa identity. Although traits are linked to taxa, their composition can shift through changes in relative abundances that may leave taxonomic composition largely unchanged (De Bello et al. 2007). The predominance of negative trends in ecological trait β‐diversity across spatial stress gradients indicates a tendency toward functional homogenization, likely reflecting convergence in ecological and habitat preferences under persistent or intense anthropogenic pressure (Sinclair, Welti, et al. 2024), particularly in relation to temperature change and land cover alteration. Conversely, differentiation in trait composition was common only under low anthropogenic stress, suggesting that even modest anthropogenic pressures can constrain trait diversity (Gutiérrez‐Cánovas et al. 2015). These findings highlight that trait‐based metrics, especially those based on ecological preferences, are more sensitive indicators of anthropogenic change than taxonomic approaches alone. Detecting trait homogenization or differentiation can offer an earlier or more nuanced signal of biodiversity responses to anthropogenic stress, emphasizing their value for conservation monitoring and assessment.

The contribution of additions and subtractions to changes in β‐diversity also varied along spatial gradients of anthropogenic stress. Additions of taxa and traits promoted differentiation at lower levels of warming and land cover pressure, but shifted toward homogenization as these stressors intensified. Subtractions showed the inverse pattern, contributing to homogenization at lower stress and differentiation at higher stress levels, although with relatively low explanatory power of our models indicating that these responses were context‐dependent. In general, differentiation was linked to additions of sensitive taxa (e.g., Ephemeroptera, Plecoptera, Trichoptera), diversification of feeding groups and microhabitat preferences, and subtractions of tolerant groups with slow life cycles (e.g., Hirudinea and Oligochaeta). These compositional shifts likely reflect improvements in habitat conditions (Gillmann et al. 2023; Schürings et al. 2025). In contrast, the opposite set of trait responses were related to homogenization, which is consistent with higher anthropogenic stress levels. These patterns suggest that the direction of β‐diversity change depends less on whether taxa or traits are being added or subtracted, and more on which specific traits are involved. The direction of change—differentiation versus homogenization—depends strongly on the stress levels and the trait composition of the communities, highlighting how functional composition mediates community responses to anthropogenic change (Gossner et al. 2023; Rivera et al. 2023; Rumschlag et al. 2023).

**FIGURE 7:**
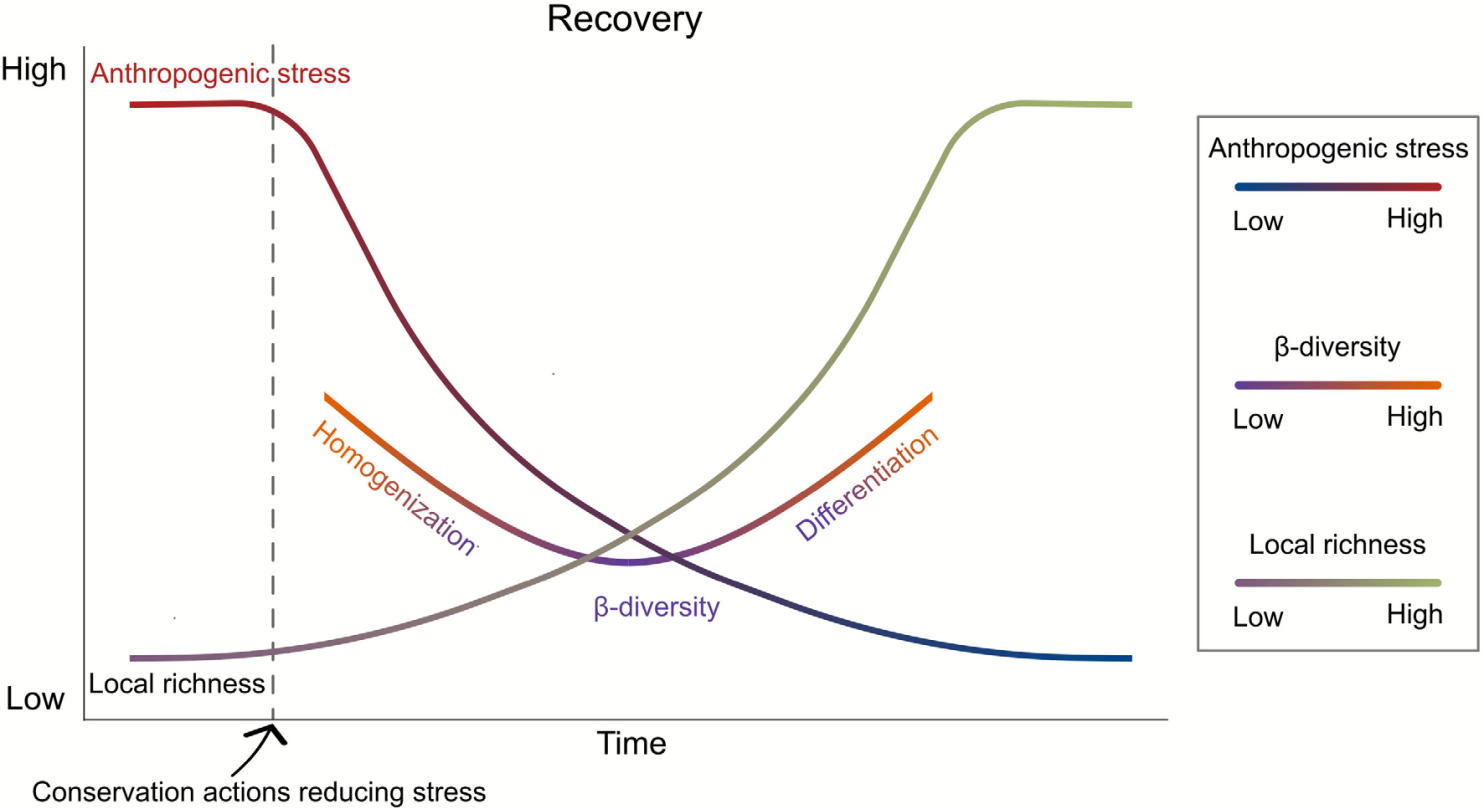
Conceptual diagram of hypothesized β‐diversity responses during recovery. Following the implementation of conservation actions, anthropogenic stress is expected to decrease while local richness increases. This might result in an initial decrease of β‐diversity when stress levels are still relatively high, and a later increase of β‐diversity when stress levels are lower and local richness is higher. While both taxonomic and trait β‐diversity may exhibit similar overall patterns, the higher sensitivity of trait β‐diversity is expected to reveal earlier or more nuanced shifts along the stress gradient.

Both taxonomic and trait β‐diversity responded asymmetrically along spatial gradients of anthropogenic stress, underscoring the complex and dynamic nature of community recovery. Notably, a general trend of local recovery—such as increases in richness—did not necessarily correspond with increased β‐diversity. This asymmetry persisted even among recovering communities, indicating that both homogenization and differentiation can emerge as outcomes of the recovery process. Such discrepancy between local and regional trends may stem from persistent or unaddressed stressors that continue to shape community composition (Aspin et al. 2018; Baker et al. 2025). Since conservation actions typically target only a subset of stressors (Haase et al. 2025), stress release tends to be a partial and gradual process. Recovery is not necessarily a linear process (Everaert et al. 2013), and increasing β‐diversity does not have to be a desirable conservation outcome (Socolar et al. 2016; Zhai et al. 2023). Although our analyses focused on β‐diversity trends along spatial gradients of anthropogenic stress, we hypothesize similar β‐diversity responses along temporal gradients of anthropogenic stress that reflect community recovery (i.e., decreasing stress following conservation actions). During recovery, communities may initially homogenize, reflected by a decline of β‐diversity at still high but declining stress levels. This likely represents the reassembly of communities dominated by generalist or resilient taxa. However, once stress is sufficiently reduced, β‐diversity increases again, indicating differentiation due to the establishment of more taxonomically and functionally distinct communities (Figure 7). Testing this hypothesis in future studies would significantly advance our understanding of biodiversity recovery dynamics.

Our findings of asymmetric responses of β‐diversity along spatial stress gradient, as well as differential responses of taxonomic‐ and trait‐based metrics, carry significant implications for conservation legislation that includes assessments of conservation action success, such as the Kunming‐Montreal Global Biodiversity Framework or the EU Nature Restoration Regulation. Conservation actions aim not only to recover species richness but also to reestablish the complexity and functionality of ecosystems (Leuzinger and Rewald 2021; Tickner et al. 2020). For example, under initially high stress levels, conservation actions might first lead to increased average occupancy of low‐abundance taxa, resulting in higher local richness but reduced β‐diversity. While increases in richness represent a critical aspect of community reassembly, they may be accompanied by functionally simplified communities dominated by generalist species. In contrast, under sufficiently low stress conditions, conservation actions result in increases in both local richness and β‐diversity—reflecting more heterogeneous and compositionally distinct communities. These findings challenge conventional conservation goals by highlighting the need for adaptive strategies for restoring ecosystem functions and services. Conservation outcomes depend on complex interactions among stressors and community composition. Thus, conservation targets must be realistic, context‐dependent, and recognize dynamic recovery trajectories. By acknowledging the asymmetrical nature of β‐diversity responses to changes in spatial anthropogenic stress levels, researchers and managers can more effectively assess progress towards conservation targets and provide recommendations that promote long‐term ecosystem structural and functional recovery.

## Author Contributions

**Daniela Cortés‐Guzmán:** conceptualization, methodology, data curation, investigation, validation, formal analysis, visualization, writing ‐ original draft, writing ‐ review and editing. **Diana E. Bowler:** methodology, validation, formal analysis, writing ‐ review and editing. **Marie Anne Eurie Forio:** validation, data curation, writing ‐ review and editing. **Peter Goethals:** validation, writing ‐ review and editing, data curation. **Ioannis Karaouzas:** validation, writing ‐ review and editing, data curation. **Ariane Moulinec:** validation, writing ‐ review and editing, data curation. **James S. Sinclair:** validation, writing ‐ review and editing, data curation, methodology. **Rudy Vannevel:** writing ‐ review and editing, validation, data curation. **Peter Haase:** conceptualization, investigation, validation, supervision, funding acquisition, writing ‐ review and editing. **Ellen A.R. Welti:** conceptualization, investigation, writing ‐ review and editing, supervision.

## Funding

This work was supported by EU Horizon 2020 project eLTER PLUS, 871128. Deutsche Forschungsgemeinschaft, 426547801. Frontiers Research Foundation. Umweltbundesamt, 3721242030. FAZIT Stiftung.

## Conflicts of Interest

The authors declare no conflicts of interest.

## Supporting information


**Figure S1:** Data distribution of anthropogenic drivers analyzed in our study across basins.
**Figure S2:**. Residual variation of taxonomic, biological, and ecological trait β‐diversity models against data descriptors (mean and maximum distance between sampling sites in a basin, number of sampling sites, and length of the time series). Residual variation was also tested for spatial and temporal autocorrelation with a Moran's I test and a Durbin‐Watson test on the residuals and the midpoint coordinates of the basins (for spatial autocorrelation) using the functions *testSpatialAutocorrelation* and *testTemporalAutocorrelation* from the “DHARMa” package (Hartig, 2024). Visual diagnostics and tests did not indicate residual autocorrelation or variance heterogeneity.
**Figure S3:**. Temporal trends in β‐diversity based on taxonomic composition (green lines), biological traits (purple lines), and ecological traits (orange lines) are shown alongside their basin of origin. Black dots show the middle point of the basins. Asterisks indicate significant trends in at least one of the three indices. Axes in all plots follow the same scaling and orientation as in the reference plot at the top.
**Figure S4:**. Trends in (a) taxonomic, (b) biological trait, and (c) ecological trait β‐diversity in 48 European basins. Significance of the slopes over time is indicated by asterisks. Few basins showed slopes significantly different from 0.
**Figure S5:** Predicted effects of (a) ecological quality, (b) temperature trends, and (c) land cover on long‐term trends in taxonomic (left panels), biological (middle panels), and ecological (right panels) trait β‐diversity from generalized linear mixed models. *R* ^2^ and *p*‐values are shown for each model; for land cover pressure, *p*‐values correspond to PC1 and PC2. Here, only recovering communities based on trends in local richness were analyzed. Line colors represent predicted trends across gradients of anthropogenic stress, ranging from lower stress (higher average EQR, lower temperature slope, or lower urban/higher forest cover) to higher stress (lower average EQR, higher temperature slope, or higher urban cover). Stress levels were represented by five equally spaced values along the range of each variable. For land cover, we selected values along the PC2 range while keeping PC1 constant. Solid lines represent significant interactions and dashed lines represent non‐significant interactions.
**Figure S6:**. β‐diversity trends as a function of local richness trends. Each point represents the estimated year slope for taxonomic, biological, and ecological trait β‐diversity, plotted against the corresponding slope in local richness. Temporal trends were estimated using generalized mixed models (see Main text), and relationships between β‐diversity and richness slopes were assessed through linear models. Regression estimates and *p*‐values are shown for each relationship.
**Table S1:**. Definitions and categories of biological and ecological traits used in this study.
**Table S2:**. Coefficients derived from the models relating taxonomic, biological, and ecological trait β‐diversity over time to ecological quality (EQR), temperature trend (Temp), and land cover pressure (PC1 and PC2).
**Table S3:**. Coefficients derived from the models relating taxonomic, biological, and ecological trait β‐diversity over time to ecological quality (EQR), temperature trend (Temp), and land cover pressure (PC1 and PC2). Here, only recovering communities based on trends in local richness were analyzed.

## Data Availability

All data needed to repeat our analyses are available from doi.org/10.6084/m9.figshare.29820404. Code necessary to replicate our analyses and produce the figures can be found in https://github.com/DanielaCortesGuzman/Differentiation‐and‐homogenization‐of‐European‐communities/tree/main.

## References

[gcb70716-bib-0001] Agra, J. , T. Cornelissen , A. B. Viana‐Junior , and M. Callisto . 2023. “Global Synthesis and Meta‐Analysis of Environmental Heterogeneity Effects on Freshwater Biodiversity.” Oikos 2024, no. 1: e10186. 10.1111/oik.10186.

[gcb70716-bib-0002] Aspin, T. W. H. , T. J. Matthews , K. Khamis , et al. 2018. “Drought Intensification Drives Turnover of Structure and Function in Stream Invertebrate Communities.” Ecography 41, no. 12: 1992–2004. 10.1111/ecog.03711.

[gcb70716-bib-0003] Astorga, A. , R. Death , F. Death , R. Paavola , M. Chakraborty , and T. Muotka . 2014. “Habitat Heterogeneity Drives the Geographical Distribution of Beta Diversity: The Case of N Ew Z Ealand Stream Invertebrates.” Ecology and Evolution 4, no. 13: 2693–2702. 10.1002/ece3.1124. 25077020 PMC4113293

[gcb70716-bib-0004] Baker, N. J. , F. Pilotto , E. A. R. Welti , D. Osadčaja , and V. Palinauskas . 2025. “Recovery or Reorganisation? Long‐Term Increases in Riverine Taxonomic and Functional Diversity Are Confounded by Compositional Dynamics.” Hydrobiologia 852, no. 7: 1693–1715. 10.1007/s10750-024-05665-5.

[gcb70716-bib-0005] Baselga, A. 2017. “Partitioning Abundance‐Based Multiple‐Site Dissimilarity Into Components: Balanced Variation in Abundance and Abundance Gradients.” Methods in Ecology and Evolution 8: 799–808. 10.1111/2041-210X.12693.

[gcb70716-bib-0006] Biggs, C. R. , L. A. Yeager , D. G. Bolser , et al. 2020. “Does Functional Redundancy Affect Ecological Stability and Resilience? A Review and Meta‐Analysis.” Ecosphere 11, no. 7: e03184. 10.1002/ecs2.3184.

[gcb70716-bib-0007] Blowes, S. A. , B. McGill , V. Brambilla , et al. 2024. “Synthesis Reveals Approximately Balanced Biotic Differentiation and Homogenization.” Science Advances 10, no. 8: eadj9395. 10.1126/sciadv.adj9395. 38381832 PMC10881054

[gcb70716-bib-0008] Bonada, N. , N. Prat , V. H. Resh , and B. Statzner . 2006. “Developments in Aquatic Insect Biomonitoring: A Comparative Analysis of Recent Approaches.” Annual Review of Entomology 51, no. 1: 495–523. 10.1146/annurev.ento.51.110104.151124. 16332221

[gcb70716-bib-0009] Bowler, D. E. , D. Eichenberg , K. Conze , et al. 2021. “Winners and Losers Over 35 Years of Dragonfly and Damselfly Distributional Change in Germany.” Diversity and Distributions 27, no. 8: 1353–1366. 10.1111/ddi.13274.

[gcb70716-bib-0010] Brooks, M. , B. Bolker , K. Kristensen , M. Maechler , and A. Magnusson . 2024. “glmmTMB: Generalized Linear Mixed Models Using Template Model Builder (Version 1.1.10) [Computer Software].” https://github.com/glmmTMB/glmmTMB.

[gcb70716-bib-0011] Burns, J. H. , and S. Y. Strauss . 2011. “More Closely Related Species Are More Ecologically Similar in an Experimental Test.” Proceedings of the National Academy of Sciences 108, no. 13: 5302–5307. 10.1073/pnas.1013003108. PMC3069184 21402914

[gcb70716-bib-0012] Cano‐Barbacil, C. , D. E. Bowler , G. A. Ballesteros‐Pelegrín , et al. 2025. “Almost Seven Decades of Coastal Bird Community Recovery Across Three European Seas.” Global Change Biology 31, no. 11: e70623. 10.1111/gcb.70623. 41292494 PMC12648369

[gcb70716-bib-0013] Cavalcante, L. L. , V. S. Daga , R. R. Braga , and A. A. Padial . 2023. “Functional Homogenization in Aquatic Ecosystems: A Review and Framework Proposal.” Hydrobiologia 850, no. 6: 1283–1302. 10.1007/s10750-022-04919-4.

[gcb70716-bib-0014] Chase, J. M. 2003. “Community Assembly: When Should History Matter?” Oecologia 136, no. 4: 489–498. 10.1007/s00442-003-1311-7. 12836009

[gcb70716-bib-0015] Chase, J. M. 2007. “Drought Mediates the Importance of Stochastic Community Assembly.” Proceedings of the National Academy of Sciences 104, no. 44: 17430–17434. 10.1073/pnas.0704350104. PMC2077273 17942690

[gcb70716-bib-0016] Chase, J. M. , B. J. McGill , P. L. Thompson , et al. 2019. “Species Richness Change Across Spatial Scales.” Oikos 128, no. 8: 1079–1091. 10.1111/oik.05968.

[gcb70716-bib-0017] Cheng, L. , S. Blanchet , G. Loot , et al. 2014. “Temporal Changes in the Taxonomic and Functional Diversity of Fish Communities in Shallow Chinese Lakes: The Effects of River–Lake Connections and Aquaculture.” Aquatic Conservation: Marine and Freshwater Ecosystems 24, no. 1: 23–34. 10.1002/aqc.2418.

[gcb70716-bib-0018] Clare, D. S. , C. Garcia , and S. G. Bolam . 2024. “Ecosystem Functioning During Biodiversity Loss and Recovery.” Oikos 2024, no. 9: e10154. 10.1111/oik.10154.

[gcb70716-bib-0019] Copernicus Climate Change Service, Climate Data Store . 2019. “Land Cover Classification Gridded Maps From 1992 to Present Derived From Satellite Observation.”

[gcb70716-bib-0020] Cornes, R. C. , G. van der Schrier , E. J. M. van den Besselaar , and P. D. Jones . 2018. “An Emsemble Version of the E‐OBS Temperature and Precipitation Data Sets.” Journal of Geophysical Research: Atmospheres 123: 9391–9409. 10.1029/2017JD028200.

[gcb70716-bib-0021] Cortés‐Guzmán, D. , D. E. Bowler , and P. Haase . 2024. “Spatial and Temporal Effects of Heat Waves on the Diversity of European Stream Invertebrate Communities.” Science of the Total Environment 953: 176229. 10.1016/j.scitotenv.2024.176229. 39270857

[gcb70716-bib-0022] Daleo, P. , J. Alberti , E. J. Chaneton , et al. 2023. “Environmental Heterogeneity Modulates the Effect of Plant Diversity on the Spatial Variability of Grassland Biomass.” Nature Communications 14, no. 1: 1809. 10.1038/s41467-023-37395-y. PMC10066197 37002217

[gcb70716-bib-0023] Davey, C. M. , D. E. Chamberlain , S. E. Newson , D. G. Noble , and A. Johnston . 2012. “Rise of the Generalists: Evidence for Climate Driven Homogenization in Avian Communities.” Global Ecology and Biogeography 21, no. 5: 568–578. 10.1111/j.1466-8238.2011.00693.x.

[gcb70716-bib-0024] Dawson, S. K. , C. P. Carmona , M. González‐Suárez , et al. 2021. “The Traits of “Trait Ecologists”: An Analysis of the Use of Trait and Functional Trait Terminology.” Ecology and Evolution 11, no. 23: 16434–16445. 10.1002/ece3.8321. 34938447 PMC8668725

[gcb70716-bib-0025] De Bello, F. , Z. Botta‐Dukát , J. Lepš , and P. Fibich . 2021. “Towards a More Balanced Combination of Multiple Traits When Computing Functional Differences Between Species.” Methods in Ecology and Evolution 12, no. 3: 443–448. 10.1111/2041-210X.13537.

[gcb70716-bib-0026] De Bello, F. , J. Lepš , S. Lavorel , and M. Moretti . 2007. “Importance of Species Abundance for Assessment of Trait Composition: An Example Based on Pollinator Communities.” Community Ecology 8, no. 2: 163–170. 10.1556/ComEc.8.2007.2.3.

[gcb70716-bib-0027] De Los Santos, C. B. , D. Krause‐Jensen , T. Alcoverro , et al. 2019. “Recent Trend Reversal for Declining European Seagrass Meadows.” Nature Communications 10, no. 1: 3356. 10.1038/s41467-019-11340-4. PMC6659699 31350407

[gcb70716-bib-0028] Dormann, C. , O. Schweiger , I. Augenstein , et al. 2007. “Effects of Landscape Structure and Land‐Use Intensity on Similarity of Plant and Animal Communities.” Global Ecology and Biogeography 16: 774–787. 10.1111/j.1466-8238.2007.00344.x.

[gcb70716-bib-0029] Dornelas, M. , N. J. Gotelli , B. McGill , et al. 2014. “Assemblage Time Series Reveal Biodiversity Change but Not Systematic Loss.” Science 344, no. 6181: 296–299. 10.1126/science.1248484. 24744374

[gcb70716-bib-0030] Dornelas, M. , N. J. Gotelli , H. Shimadzu , F. Moyes , A. E. Magurran , and B. J. McGill . 2019. “A Balance of Winners and Losers in the Anthropocene.” Ecology Letters 22, no. 5: 847–854. 10.1111/ele.13242. 30874368

[gcb70716-bib-0031] Ekroos, J. , J. Heliölä , and M. Kuussaari . 2010. “Homogenization of Lepidopteran Communities in Intensively Cultivated Agricultural Landscapes.” Journal of Applied Ecology 47, no. 2: 459–467. 10.1111/j.1365-2664.2009.01767.x.

[gcb70716-bib-0032] Everaert, G. , I. S. Pauwels , P. Boets , et al. 2013. “Model‐Based Evaluation of Ecological Bank Design and Management in the Scope of the European Water Framework Directive.” Ecological Engineering 53: 144–152. 10.1016/j.ecoleng.2012.12.034.

[gcb70716-bib-0033] Floury, M. , P. Usseglio‐Polatera , M. Ferreol , C. Delattre , and Y. Souchon . 2013. “Global Climate Change in Large European Rivers: Long‐Term Effects on Macroinvertebrate Communities and Potential Local Confounding Factors.” Global Change Biology 19, no. 4: 1085–1099. 10.1111/gcb.12124. 23504886

[gcb70716-bib-0034] Giakoumis, T. , and N. Voulvoulis . 2018. “The Transition of EU Water Policy Towards the Water Framework Directive's Integrated River Basin Management Paradigm.” Environmental Management 62, no. 5: 819–831. 10.1007/s00267-018-1080-z. 29987347 PMC6208820

[gcb70716-bib-0035] Gillmann, S. M. , D. Hering , and A. W. Lorenz . 2023. “Habitat Development and Species Arrival Drive Succession of the Benthic Invertebrate Community in Restored Urban Streams.” Environmental Sciences Europe 35, no. 1: 49. 10.1186/s12302-023-00756-x.

[gcb70716-bib-0036] Gossner, M. M. , F. Menzel , and N. K. Simons . 2023. “Less Overall, but More of the Same: Drivers of Insect Population Trends Lead to Community Homogenization.” Biology Letters 19, no. 3: 20230007. 10.1098/rsbl.2023.0007. 36987614 PMC10050920

[gcb70716-bib-0037] Gutiérrez‐Cánovas, C. , D. Sánchez‐Fernández , J. Velasco , A. Millán , and N. Bonada . 2015. “Similarity in the Difference: Changes in Community Functional Features Along Natural and Anthropogenic Stress Gradients.” Ecology 96, no. 9: 2458–2466. 10.1890/14-1447.1. 26594702

[gcb70716-bib-0038] Haase, P. , D. E. Bowler , N. J. Baker , et al. 2023. “The Recovery of European Freshwater Biodiversity Has Come to a Halt.” Nature 620: 582–588. 10.1038/s41586-023-06400-1. 37558875 PMC10432276

[gcb70716-bib-0039] Haase, P. , D. Cortés‐Guzmán , F. He , et al. 2025. “Successes and Failures of Conservation Actions to Halt Global River Biodiversity Loss.” Nature Reviews Biodiversity 1: 104–118. 10.1038/s44358-024-00012-x.

[gcb70716-bib-0040] Hawkins, C. P. , H. Mykrä , J. Oksanen , and J. J. Vander Laan . 2015. “Environmental Disturbance Can Increase Beta Diversity of Stream Macroinvertebrate Assemblages.” Global Ecology and Biogeography 24, no. 4: 483–494. 10.1111/geb.12254.

[gcb70716-bib-0041] Hillebrand, H. , B. Blasius , E. T. Borer , et al. 2018. “Biodiversity Change Is Uncoupled From Species Richness Trends: Consequences for Conservation and Monitoring.” Journal of Applied Ecology 55, no. 1: 169–184. 10.1111/1365-2664.12959.

[gcb70716-bib-0042] Huber, P. , S. Metz , F. Unrein , G. Mayora , H. Sarmento , and M. Devercelli . 2020. “Environmental Heterogeneity Determines the Ecological Processes That Govern Bacterial Metacommunity Assembly in a Floodplain River System.” ISME Journal 14, no. 12: 2951–2966. 10.1038/s41396-020-0723-2. 32719401 PMC7784992

[gcb70716-bib-0043] Hutchings, J. A. , S. H. M. Butchart , B. Collen , M. K. Schwartz , and R. S. Waples . 2012. “Red Flags: Correlates of Impaired Species Recovery.” Trends in Ecology & Evolution 27, no. 10: 542–546. 10.1016/j.tree.2012.06.005. 22784411

[gcb70716-bib-0044] Johnson, T. F. , N. J. B. Isaac , A. Paviolo , and M. González‐Suárez . 2021. “Handling Missing Values in Trait Data.” Global Ecology and Biogeography 30, no. 1: 51–62. 10.1111/geb.13185.

[gcb70716-bib-0045] Karaouzas, I. , K. C. Gritzalis , and N. Skoulikidis . 2007. “Land Use Effects on Macroinvertebrate Assemblages and Stream Quality Along an Agricultural River Basin.” Fresenius Environmental Bulletin 16, no. 6: 645–653.

[gcb70716-bib-0046] Laliberté, E. , P. Legendre , and B. Shipley . 2014. “FD: Measuring Functional Diversity From Multiple Traits, and Other Tools for Functional Ecology(Version 1.0‐12.1.) [R].” 10.1890/08-2244.1 20380219

[gcb70716-bib-0047] Lavorel, S. , K. Grigulis , S. McIntyre , et al. 2008. “Assessing Functional Diversity in the Field – Methodology Matters!” Functional Ecology 22, no. 1: 134–147. 10.1111/j.1365-2435.2007.01339.x.

[gcb70716-bib-0048] Leibold, M. A. , M. Holyoak , N. Mouquet , et al. 2004. “The Metacommunity Concept: A Framework for Multi‐Scale Community Ecology.” Ecology Letters 7: 601–613. 10.1111/j.1461-0248.2004.00608.x.

[gcb70716-bib-0049] Leroy, F. , J. Reif , Z. Vermouzek , et al. 2024. “Decomposing Biodiversity Change to Processes of Extinction, Colonization, and Recurrence Across Scales.” Ecography 2024, no. 2: e06995. 10.1111/ecog.06995.

[gcb70716-bib-0050] Leuzinger, S. , and B. Rewald . 2021. “The Who or the How? Species vs. Ecosystem Function Priorities in Conservation Ecology.” Frontiers in Plant Science 12: 758413. 10.3389/fpls.2021.758413. 34795686 PMC8593376

[gcb70716-bib-0051] Li, L. , B. Zheng , and L. Liu . 2010. “Biomonitoring and Bioindicators Used for River Ecosystems: Definitions, Approaches and Trends.” Procedia Environmental Sciences 2: 1510–1524. 10.1016/j.proenv.2010.10.164.

[gcb70716-bib-0052] Liang, J. , Z. Ding , G. Lie , Z. Zhou , Z. Zhang , and H. Hu . 2025. “Climate‐Driven Environmental Filtering Determines Hump‐Shaped Elevational Pattern of Seed Plant Beta Diversity in the Central Himalayas.” Plant Diversity 47, no. 2: 264–272. 10.1016/j.pld.2024.07.011. 40182491 PMC11963152

[gcb70716-bib-0053] Lyche Solheim, A. , J.‐E. Thrane , S. Mentzel , and S. J. Moe . 2025. “Harmonised Biological Indicators for Rivers and Lakes: Towards European Assessment of Temporal Trends in Ecological Quality.” Ecological Indicators 171: 113207. 10.1016/j.ecolind.2025.113207.

[gcb70716-bib-0054] Magurran, A. E. , M. Dornelas , F. Moyes , N. J. Gotelli , and B. McGill . 2015. “Rapid Biotic Homogenization of Marine Fish Assemblages.” Nature Communications 6, no. 1: 8405. 10.1038/ncomms9405. PMC4598618 26400102

[gcb70716-bib-0055] Marr, S. M. , J. D. Olden , F. Leprieur , et al. 2013. “A Global Assessment of Freshwater Fish Introductions in Mediterranean‐Climate Regions.” Hydrobiologia 719, no. 1: 317–329. 10.1007/s10750-013-1486-9.

[gcb70716-bib-0056] O'Briain, R. 2019. “Climate Change and European Rivers: An Eco‐Hydromorphological Perspective.” Ecohydrology 12, no. 5: e2099. 10.1002/eco.2099.

[gcb70716-bib-0057] Outhwaite, C. L. , R. D. Gregory , R. E. Chandler , B. Collen , and N. J. B. Isaac . 2020. “Complex Long‐Term Biodiversity Change Among Invertebrates, Bryophytes and Lichens.” Nature Ecology & Evolution 4, no. 3: 384–392. 10.1038/s41559-020-1111-z. 32066888

[gcb70716-bib-0058] Palacio, F. X. , C. T. Callaghan , P. Cardoso , et al. 2022. “A Protocol for Reproducible Functional Diversity Analyses.” Ecography 2022, no. 11: e06287. 10.1111/ecog.06287.

[gcb70716-bib-0059] Petsch, D. K. 2016. “Causes and Consequences of Biotic Homogenization in Freshwater Ecosystems: Biotic Homogenization of Freshwater Systems.” International Review of Hydrobiology 101, no. 3–4: 113–122. 10.1002/iroh.201601850.

[gcb70716-bib-0060] Poyatos, R. , O. Sus , L. Badiella , M. Mencuccini , and J. Martínez‐Vilalta . 2018. “Gap‐Filling a Spatially Explicit Plant Trait Database: Comparing Imputation Methods and Different Levels of Environmental Information.” Biogeosciences 15, no. 9: 2601–2617. 10.5194/bg-15-2601-2018.

[gcb70716-bib-0061] R Core Team . 2024. “R: A Language and Environment for Statistical Computing. R Foundation for Statistical Computing, Vienna, Austria. (Version 4.3.2‐4) [Computer Software].”. https://www.R‐project.org/.

[gcb70716-bib-0062] Rivera, J. D. , A. E. De Los Monteros , R. A. Saldaña‐Vázquez , and M. E. Favila . 2023. “Beyond Species Loss: How Anthropogenic Disturbances Drive Functional and Phylogenetic Homogenization of Neotropical Dung Beetles.” Science of the Total Environment 869: 161663. 10.1016/j.scitotenv.2023.161663. 36682564

[gcb70716-bib-0063] Rolls, R. J. , D. C. Deane , S. E. Johnson , J. Heino , M. J. Anderson , and K. E. Ellingsen . 2023. “Biotic Homogenisation and Differentiation as Directional Change in Beta Diversity: Synthesising Driver–Response Relationships to Develop Conceptual Models Across Ecosystems.” Biological Reviews 98: brv.12958. 10.1111/brv.12958. 37072381

[gcb70716-bib-0064] Rumschlag, S. L. , M. B. Mahon , D. K. Jones , et al. 2023. “Density Declines, Richness Increases, and Composition Shifts in Stream Macroinvertebrates.” Science Advances 9, no. 18: eadf4896. 10.1126/sciadv.adf4896. 37134169 PMC10156106

[gcb70716-bib-0065] Schmidt‐Kloiber, A. , and D. Hering . 2015. “www.freshwaterecology.info—An Online Tool That Unifies, Standardises and Codifies More Than 20,000 European Freshwater Organisms and Their Ecological Preferences.” Ecological Indicators 53: 271–282. 10.1016/j.ecolind.2015.02.007.

[gcb70716-bib-0066] Schürings, C. , W. Kaijser , S. M. Gillmann , et al. 2025. “Drivers of Recovery and Degradation of Riverine Benthic Invertebrate Communities: A Germany‐Wide Analysis.” Ecological Processes 14, no. 1: 30. 10.1186/s13717-025-00593-1.

[gcb70716-bib-0067] Sinclair, J. S. , R. Stubbington , R. B. Schäfer , et al. 2024. “Ecological but Not Biological Traits of European Riverine Invertebrates Respond Consistently to Anthropogenic Impacts.” Global Ecology and Biogeography 33, no. 12: e13931. 10.1111/geb.13931.

[gcb70716-bib-0068] Sinclair, J. S. , E. A. R. Welti , F. Altermatt , et al. 2024. “Multi‐Decadal Improvements in the Ecological Quality of European Rivers Are Not Consistently Reflected in Biodiversity Metrics.” Nature Ecology & Evolution 8: 430–441. 10.1038/s41559-023-02305-4. 38278985

[gcb70716-bib-0069] Socolar, J. B. , J. J. Gilroy , W. E. Kunin , and D. P. Edwards . 2016. “How Should Beta‐Diversity Inform Biodiversity Conservation?” Trends in Ecology & Evolution 31, no. 1: 67–80. 10.1016/j.tree.2015.11.005. 26701706

[gcb70716-bib-0070] Su, G. , M. Logez , J. Xu , S. Tao , S. Villéger , and S. Brosse . 2021. “Human Impacts on Global Freshwater Fish Biodiversity.” Science 371, no. 6531: 835–838. 10.1126/science.abd3369. 33602854

[gcb70716-bib-0071] Tatsumi, S. , R. Iritani , and M. W. Cadotte . 2021. “Temporal Changes in Spatial Variation: Partitioning the Extinction and Colonisation Components of Beta Diversity.” Ecology Letters 24, no. 5: 1063–1072. 10.1111/ele.13720. 33715273

[gcb70716-bib-0072] Tatsumi, S. , R. Iritani , and M. W. Cadotte . 2022. “Partitioning the Temporal Changes in Abundance‐Based Beta Diversity Into Loss and Gain Components.” Methods in Ecology and Evolution 13: 2042–2048. 10.1111/2041-210X.13921.

[gcb70716-bib-0073] Theodoropoulos, C. , and I. Karaouzas . 2021. “Climate Change and the Future of Mediterranean Freshwater Macroinvertebrates: A Model‐Based Assessment.” Hydrobiologia 848, no. 21: 5033–5050. 10.1007/s10750-021-04691-x.

[gcb70716-bib-0074] Tickner, D. , J. J. Opperman , R. Abell , et al. 2020. “Bending the Curve of Global Freshwater Biodiversity Loss: An Emergency Recovery Plan.” Bioscience 70, no. 4: 330–342. 10.1093/biosci/biaa002. 32284631 PMC7138689

[gcb70716-bib-0075] Tonkin, J. D. , J. Heino , and F. Altermatt . 2018. “Metacommunities in River Networks: The Importance of Network Structure and Connectivity on Patterns and Processes.” Freshwater Biology 63, no. 1: 1–5. 10.1111/fwb.13045.

[gcb70716-bib-0076] Usseglio‐Polatera, P. , M. Bournaud , P. Richoux , and H. Tachet . 2000. “Biological and Ecological Traits of Benthic Freshwater Macroinvertebrates: Relationships and Definition of Groups With Similar Traits.” Freshwater Biology 43: 17–205. 10.1046/j.1365-2427.2000.00535.x.

[gcb70716-bib-0077] Violle, C. , M. Navas , D. Vile , et al. 2007. “Let the Concept of Trait Be Functional!” Oikos 116, no. 5: 882–892. 10.1111/j.0030-1299.2007.15559.x.

[gcb70716-bib-0078] Voulvoulis, N. , K. D. Arpon , and T. Giakoumis . 2017. “The EU Water Framework Directive: From Great Expectations to Problems With Implementation.” Science of the Total Environment 575: 358–366. 10.1016/j.scitotenv.2016.09.228. 27744201

[gcb70716-bib-0079] Water Framework Directive (WFD) . 2000. “Directive 2000/60/EC of the European Parliament and of the Council of 23 October 2000 Establishing a Framework for Community Action in the Field of Water Policy.” Official Journal of the European Communities 327: 0001–0073.

[gcb70716-bib-0080] Weisner, O. , J. Arle , L. Liebmann , et al. 2022. “Three Reasons Why the Water Framework Directive (WFD) Fails to Identify Pesticide Risks.” Water Research 208: 117848. 10.1016/j.watres.2021.117848. 34781190

[gcb70716-bib-0081] Welti, E. A. R. , D. E. Bowler , J. S. Sinclair , et al. 2024. “Time Series of Freshwater Macroinvertebrate Abundances and Site Characteristics of European Streams and Rivers.” Scientific Data 11: 601. 38849407 10.1038/s41597-024-03445-3 PMC11161585

[gcb70716-bib-0082] Westwood, A. , E. Reuchlin‐Hugenholtz , and D. M. Keith . 2014. “Re‐Defining Recovery: A Generalized Framework for Assessing Species Recovery.” Biological Conservation 172: 155–162. 10.1016/j.biocon.2014.02.031.

[gcb70716-bib-0083] Xu, W.‐B. , S. A. Blowes , V. Brambilla , et al. 2023. “Regional Occupancy Increases for Widespread Species but Decreases for Narrowly Distributed Species in Metacommunity Time Series.” Nature Communications 14, no. 1: 1463. 10.1038/s41467-023-37127-2. PMC10020147 36927847

[gcb70716-bib-0084] Zhai, M. , J. Bojková , D. Němejcová , M. Polášek , V. Syrovátka , and M. Horsák . 2023. “Climatically Promoted Taxonomic Homogenization of Macroinvertebrates in Unaffected Streams Varies Along the River Continuum.” Scientific Reports 13, no. 1: 6292. 10.1038/s41598-023-32806-y. 37072510 PMC10113374

