# Supplementary material for: Recovering European River Invertebrate Communities Homogenize or Differentiate Depending on Anthropogenic Stress
Source: Glob Chang Biol. 2026 Jan 22;32(1):e70716. doi: 10.1111/gcb.70716 (PMC12824827; doi:10.1111/gcb.70716)
Supplement: Supplementary file 1 — Figure S1: Data distribution of anthropogenic drivers analyzed in our study across basins. Figure S2:. Residual variation of taxonomic, biological, and ecological trait β‐diversity models against data descriptors (mean and maximum distance between sampling sites in a basin, number of sampling sites, and length of the time series). Residual variation was also tested for spatial and temporal autocorrelation with a Moran's I test and a Durbin‐Watson test on the residuals and the midpoint coordinates of the basins (for spatial autocorrelation) using the functions testSpatialAutocorrelation and testTemporalAutocorrelation from the “DHARMa” package (Hartig, 2024). Visual diagnostics and tests did not indicate residual autocorrelation or variance heterogeneity. Figure S3:. Temporal trends in β‐diversity based on taxonomic composition (green lines), biological traits (purple lines), and ecological traits (orange lines) are shown alongside their basin of origin. Black dots show the middle point of the basins. Asterisks indicate significant trends in at least one of the three indices. Axes in all plots follow the same scaling and orientation as in the reference plot at the top. Figure S4:. Trends in (a) taxonomic, (b) biological trait, and (c) ecological trait β‐diversity in 48 European basins. Significance of the slopes over time is indicated by asterisks. Few basins showed slopes significantly different from 0. Figure S5: Predicted effects of (a) ecological quality, (b) temperature trends, and (c) land cover on long‐term trends in taxonomic (left panels), biological (middle panels), and ecological (right panels) trait β‐diversity from generalized linear mixed models. R 2 and p‐values are shown for each model; for land cover pressure, p‐values correspond to PC1 and PC2. Here, only recovering communities based on trends in local richness were analyzed. Line colors represent predicted trends across gradients of anthropogenic stress, ranging from lower stress (higher average EQ [file GCB-32-e70716-s001.docx]

**Recovering European river invertebrate communities homogenize or differentiate depending on anthropogenic stress**

Daniela Cortés-Guzmán, Diana E. Bowler, Marie Anne Eurie Forio, Peter Goethals, Ioannis Karaouzas, Ariane Moulinec, James S. Sinclair, Rudy Vannevel, Peter Haase, Ellen A.R. Welti

# Supplementary Information


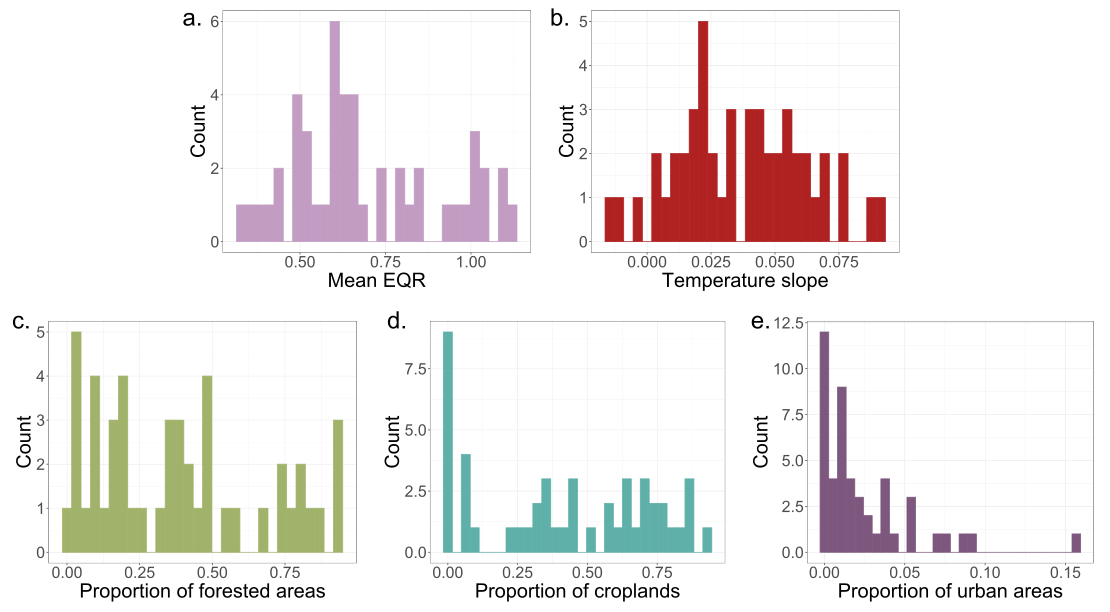


**Figure S1.** Data distribution of anthropogenic drivers analyzed in our study across basins.


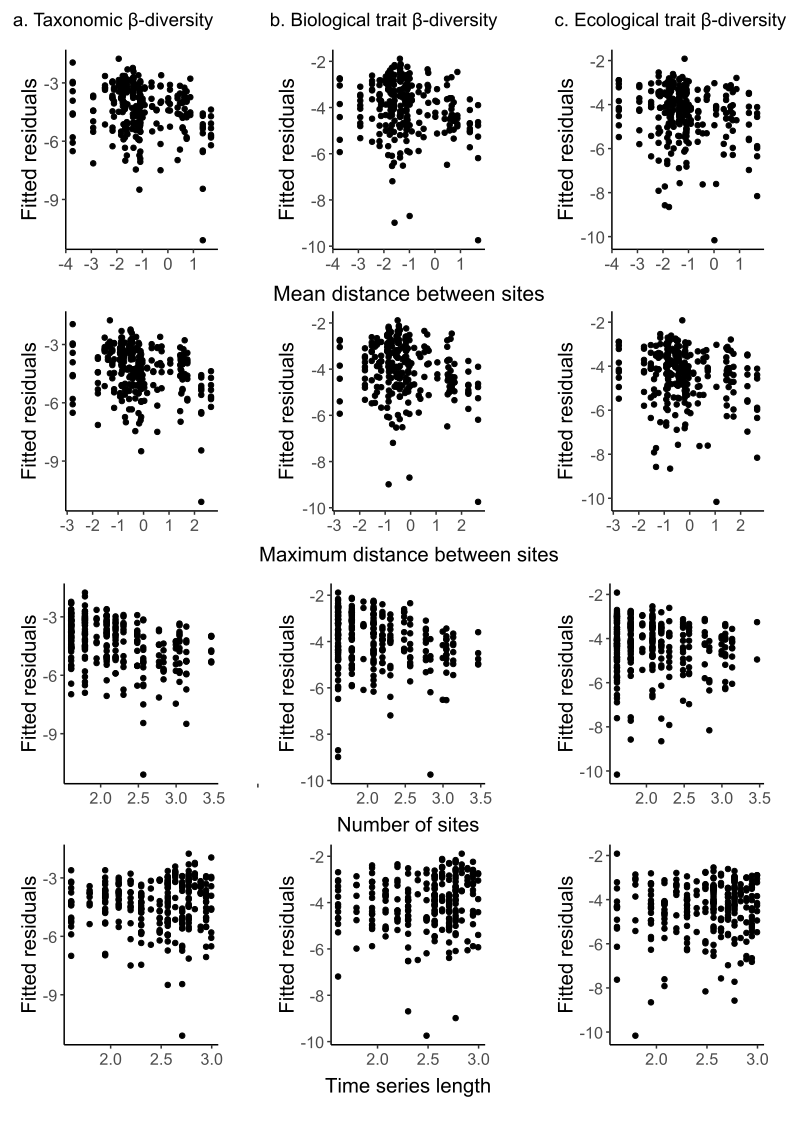


**Figure S2**. Residual variation of taxonomic, biological, and ecological trait ꞵ-diversity models against data descriptors (mean and maximum distance between sampling sites in a basin, number of sampling sites, and length of the time series). Residual variation was also tested for spatial and temporal autocorrelation with a Moran’s I test and a Durbin-Watson test on the residuals and the midpoint coordinates of the basins (for spatial autocorrelation) using the functions *testSpatialAutocorrelation* and *testTemporalAutocorrelation* from the “DHARMa” package (Hartig, 2024). Visual diagnostics and tests did not indicate residual autocorrelation or variance heterogeneity.

[Hartig, F. (2024). *DHARMa: Residual Diagnostics for Hierarchical (Multi-Level / Mixed) Regression Models* (Version 0.4.7) [R]. http://florianhartig.github.io/DHARMa/](https://www.zotero.org/google-docs/?RFe9lv)

**
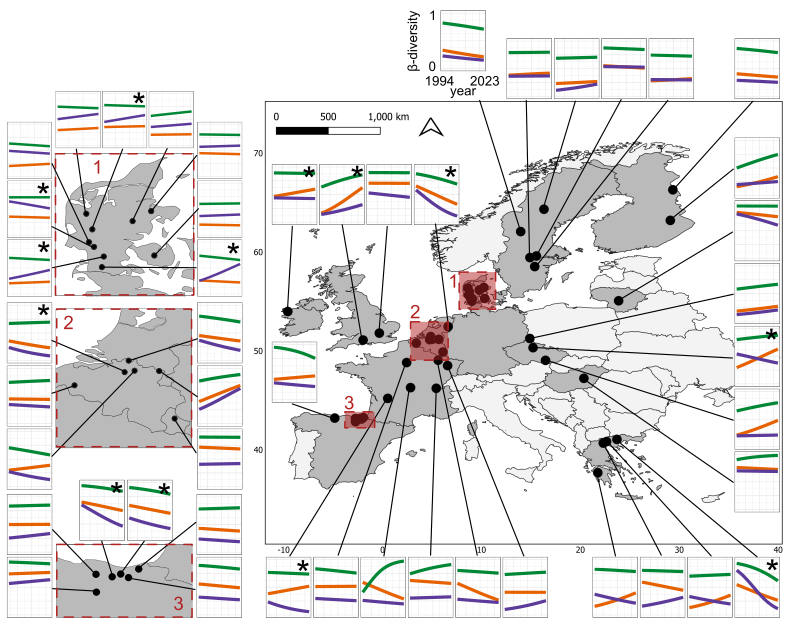
**

**Figure S3.** Temporal trends in ꞵ-diversity based on taxonomic composition (green lines), biological traits (purple lines), and ecological traits (orange lines) are shown alongside their basin of origin. Black dots show the middle point of the basins. Asterisks indicate significant trends in at least one of the three indices. Axes in all plots follow the same scaling and orientation as in the reference plot at the top.


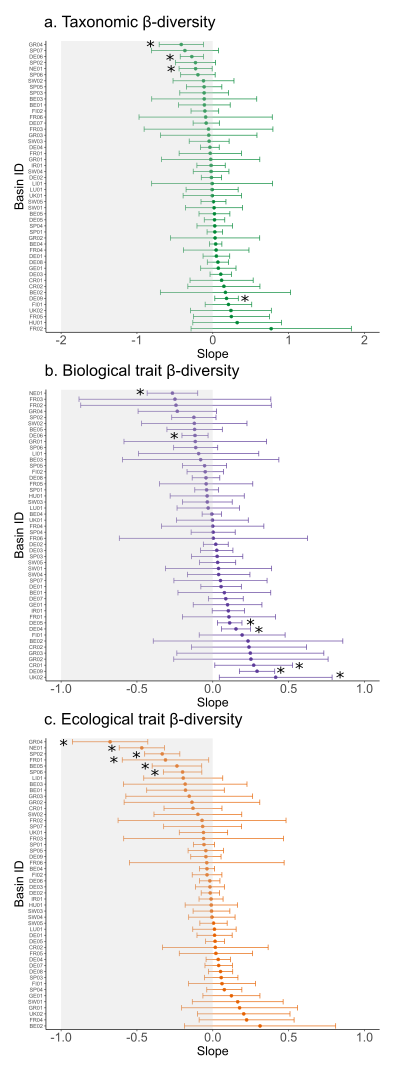


**Figure S4**. Trends in (a) taxonomic, (b) biological trait, and (c) ecological trait ꞵ-diversity in 48 European basins. Significance of the slopes over time is indicated by asterisks. Few basins showed slopes significantly different from 0.


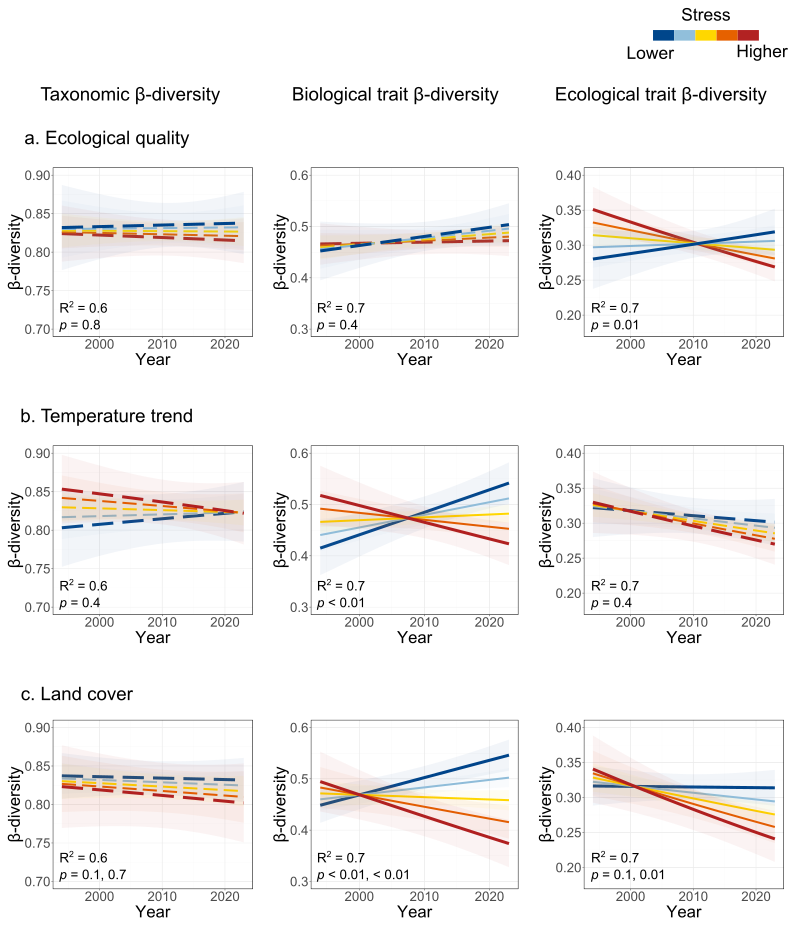


**Figure S5.** Predicted effects of (a) ecological quality, (b) temperature trends, and (c) land cover on long-term trends in taxonomic (left panels), biological (middle panels), and ecological (right panels) trait ꞵ-diversity from generalized linear mixed models. R2 and p-values are shown for each model; for land cover pressure, p-values correspond to PC1 and PC2. Here, only recovering communities based on trends in local richness were analyzed. Line colors represent predicted trends across gradients of anthropogenic stress, ranging from lower stress (higher average EQR, lower temperature slope, or lower urban/higher forest cover) to higher stress (lower average EQR, higher temperature slope, or higher urban cover). Stress levels were represented by five equally spaced values along the range of each variable. For land cover, we selected values along the PC2 range while keeping PC1 constant. Solid lines represent significant interactions and dashed lines represent non-significant interactions.


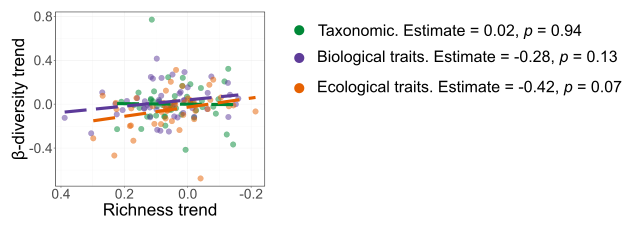


**Figure S6.** ꞵ-diversity trends as a function of local richness trends. Each point represents the estimated year slope for taxonomic, biological, and ecological trait ꞵ-diversity, plotted against the corresponding slope in local richness. Temporal trends were estimated using generalized mixed models (see Main text), and relationships between ꞵ-diversity and richness slopes were assessed through linear models. Regression estimates and *p*-values are shown for each relationship.

**Table S1**. Definitions and categories of biological and ecological traits used in this study.

| **Trait name** | **Definition** | **Categories** | **References** |
| --- | --- | --- | --- |
| Biological traits | | | |
| Aquatic stages | The life stages of a species that occur in water distinguishing eggs, larvae, nymphs and pupae, and adults. | Egg | Tachet et al., 2010 |
|  |  | Larva |  |
|  |  | Nymph |  |
|  |  | Adult |  |
| Dispersal strategy | Dispersal strategy of taxa distinguishing active or passive (e.g., drift), and aquatic or aerial (e.g., flying insects). | Aerial active | Tachet et al., 2010 |
|  |  | Aerial passive |  |
|  |  | Aquatic active |  |
|  |  | Aquatic passive |  |
| Number of reproductive cycles | The number of reproductive cycles or generations per year distinguishing species able to produce less than one generation per year (i.e., semivoltine), one generation per year (i.e., univoltine), or more than one generation per year (i.e., polyvoltine). | < 1 per year | Tachet et al., 2010 |
|  |  | 1 per year |  |
|  |  | > 1 per year |  |
| Feeding type | The feeding preference of a species distinguishing grazers (feed on algae, biofilm, living plants), shredders (feed on fallen leaves, CPOM), gatherers (feed on sedimented FPOM), active and passive filter feeders (feed on suspended POM), predators (feed on prey), miners (feed on cells of aquatic plants), xylophagous (feed on woody debris), and parasites (feed on hosts). | Grazers | Moog (1995) |
|  |  | Shredders |  |
|  |  | Gatherers |  |
|  |  | Active filter feeders |  |
|  |  | Passive filter feeders |  |
|  |  | Predators |  |
|  |  | Miners |  |
|  |  | Xylophagous |  |
|  |  | Parasites |  |
|  |  | Other |  |
| Resistance form | Specific survival strategies for unfavorable conditions distinguishing protective cocoons, diapause, dormant eggs, and housing against desiccation. | Cocoons | Tachet et al., 2010 |
|  |  | Diapause |  |
|  |  | Dormant eggs |  |
|  |  | Housing |  |
|  |  | None |  |
| Body size | The maximal size that the last aquatic stage of the taxon can reach in a large variety of (more or less favorable) environmental conditions. | < 0.25 cm | Tachet et al., 2010 |
|  |  | 0.25–0.50 cm |  |
|  |  | 0.5–1 cm |  |
|  |  | 1–2 cm |  |
|  |  | 2–4 cm |  |
|  |  | 4–8 cm |  |
|  |  | > 8 cm |  |
| Ecological traits | | | |
| Temperature preference | Temperature preference of taxa distinguishing eurythermal (broad tolerance, taxa able to live in a broad range of temperatures), psychrophilic (cold tolerant, taxa exhibiting distributions limited by water temperature upper than 15°C), and thermophilic (warm-tolerant, taxa exhibiting distributions limited by water temperature lower than 15°C). | Eurythermal | Tachet et al., 2010 |
|  |  | Psychrophilic |  |
|  |  | Thermophilic |  |
| Microhabitat preference | The preference of a species for certain microhabitats distinguishing preferences based on the grain size from argyllal (silts, loam, and clay; grain size < 0.063 mm) to lithral (coarse gravel, stones, cobbles, boulders, and bedrock; grain size > 2cm) and on the type of substrate including phytal (algae, mosses, and macrophytes) and POM (coarse and fine particulate organic matter). | Argyllal | Moog et al. (1999), Schmedtje & Colling (1996) and the AQEM Manual (AQEM consortium 2002) |
|  |  | Pelal |  |
|  |  | Psammal |  |
|  |  | Sense |  |
|  |  | Lithral |  |
|  |  | Phytal |  |
|  |  | POM |  |
|  |  | Other |  |
| Saprobity | The distribution of taxa along a gradient of contamination by organic matter distinguishing xenosaprobic species (occurring in perfectly clean waters), oligosaprobic (occurring in unpolluted to slightly polluted waters), ꞵ-mesosaprobic (occurring in moderately polluted waters), α-mesosaprobic (occurring in heavily polluted waters), and polysaprobic (occurring in extremely heavily polluted waters). | Xenosaprobic | Tachet et al., 2010 |
|  |  | Oligosaprobic |  |
|  |  | ꞵ-Mesosaprobic |  |
|  |  | α-Mesosaprobic |  |
|  |  | Polysaprobic |  |

**References**

AQEM Consortium. (2003). Manual for the Application of the AQEM System: A Comprehensive Method to Assess European Streams Using Benthic Macroinvertebrates, Developed for the Purpose of the Water Framework Directive. http://www.aqem.de/ftp/aqem_manual.zip

Moog, E. (1995). Fauna Aquatica Austriaca—A Comprehensive Species Inventory of Austrian Aquatic Organisms with Ecological Notes. Federal Ministry for Agriculture and Forestry, Wasserwirtschaftskataster Vienna: loose-leaf binder.

Moog, O., Chovanec, A., Hinteregger, H., & Römer, A. (1999). Richtlinie für die saprobiologische Gewässergütebeurteilung von Fließgewässern. Wasserwirtschaftskataster, Bundesministerium für Land- und Forstwirtschaft.

Schmedtje, U., & Colling, M. (1996). Ökologische Typisierung der aquatischen Makrofauna. Informationsberichte des Bayerischen Landesamtes für Wasserwirtschaft.

Tachet, H., Bournaud, M., Richoux, P., & Usseglio-Polatera, P. (2010). Invertébrés d’eau douce—Systématique, biologie, écologie. CNRS Editions.

**Table S2**. Coefficients derived from the models relating taxonomic, biological, and ecological trait ꞵ-diversity over time to ecological quality (EQR), temperature trend (Temp), and land cover pressure (PC1 and PC2).

| Taxonomic ꞵ-diversity | ꞵ-div = 1.54 − 0.01*Year + 0.01*EQR + 0.49*ꞵi + 0.01*Year*EQR |
| --- | --- |
|  | ꞵ-div = 1.54 − 0.01*Year + 0.05*Temp + 0.48*ꞵi − 0.01*Year*Temp |
|  | ꞵ-div = 1.54 − 0.01*Year + 0.00*PC1 − 0.02*PC2 + 0.49*ꞵi − 0.01*Year*PC1 − 0.00*PC2 |
| Biological trait ꞵ-diversity | ꞵ-div = −0.11 + 0.02*Year + 0.02*EQR + 0.52*ꞵi + 0.02*Year*EQR |
|  | ꞵ-div = −0.11 + 0.01*Year + 0.01*Temp + 0.52*ꞵi − 0.02*Year*Temp |
|  | ꞵ-div = −0.11 + 0.01*Year − 0.00*PC1 − 0.06*PC2 + 0.51*ꞵi − 0.01*Year*PC1 − 0.03*PC2 |
| Ecological trait ꞵ-diversity | ꞵ-div = −0.86 − 0.02*Year + 0.02*EQR + 0.55*ꞵi + 0.03*Year*EQR |
|  | ꞵ-div = −0.86 − 0.03*Year + 0.02*Temp + 0.54*ꞵi − 0.01*Year*Temp |
|  | ꞵ-div = −0.85 − 0.03*Year − 0.00*PC1 − 0.03*PC2 + 0.54*ꞵi − 0.01*Year*PC1 − 0.02*PC2 |

**Table S3**. Coefficients derived from the models relating taxonomic, biological, and ecological trait ꞵ-diversity over time to ecological quality (EQR), temperature trend (Temp), and land cover pressure (PC1 and PC2). Here, only recovering communities based on trends in local richness were analyzed.

| Taxonomic ꞵ-diversity | ꞵ-div = 1.55 − 0.00*Year + 0.03*EQR + 0.49*ꞵi + 0.01*Year*EQR |
| --- | --- |
|  | ꞵ-div = 1.55 − 0.01*Year + 0.03*Temp + 0.48*ꞵi − 0.02*Year*Temp |
|  | ꞵ-div = 1.57 − 0.01*Year + 0.00*PC1 − 0.03*PC2 + 0.48*ꞵi − 0.02*Year*PC1 − 0.00*PC2 |
| Biological trait ꞵ-diversity | ꞵ-div = −0.10 + 0.02*Year + 0.01*EQR + 0.53*ꞵi + 0.01*Year*EQR |
|  | ꞵ-div = −0.10 + 0.02*Year + 0.02*Temp + 0.54*ꞵi − 0.04*Year*Temp |
|  | ꞵ-div = −0.08 + 0.02*Year − 0.02*PC1 − 0.07*PC2 + 0.52*ꞵi − 0.02*Year*PC1 − 0.04*PC2 |
| Ecological trait ꞵ-diversity | ꞵ-div = −0.84 − 0.03*Year + 0.00*EQR + 0.56*ꞵi + 0.03*Year*EQR |
|  | ꞵ-div = −0.84 − 0.04*Year - 0.02*Temp + 0.56*ꞵi − 0.01*Year*Temp |
|  | ꞵ-div = −0.83 − 0.03*Year − 0.01*PC1 − 0.04*PC2 + 0.55*ꞵi − 0.01*Year*PC1 − 0.02*PC2 |
